# Functional Connectome Dynamics After Mild Traumatic Brain Injury According to Age and Sex

**DOI:** 10.3389/fnagi.2022.852990

**Published:** 2022-05-18

**Authors:** Anar Amgalan, Alexander S. Maher, Phoebe Imms, Michelle Y. Ha, Timothy A. Fanelle, Andrei Irimia

**Affiliations:** ^1^Ethel Percy Andrus Gerontology Center, Leonard Davis School of Gerontology, University of Southern California, Los Angeles, CA, United States; ^2^Corwin D. Denney Research Center, Department of Biomedical Engineering, Viterbi School of Engineering, University of Southern California, Los Angeles, CA, United States

**Keywords:** functional neuroimaging, human connectome, neurophysiology, default-mode network, attention, limbic system

## Abstract

Neural and cognitive deficits after mild traumatic brain injury (mTBI) are paralleled by changes in resting state functional correlation (FC) networks that mirror post-traumatic pathophysiology effects on functional outcomes. Using functional magnetic resonance images acquired both acutely and chronically after injury (∼1 week and ∼6 months post-injury, respectively), we map post-traumatic FC changes across 136 participants aged 19–79 (52 females), both within and between the brain’s seven canonical FC networks: default mode, dorsal attention, frontoparietal, limbic, somatomotor, ventral attention, and visual. Significant sex-dependent FC changes are identified between (A) visual and limbic, and between (B) default mode and somatomotor networks. These changes are significantly associated with specific functional recovery patterns across all cognitive domains (*p* < 0.05, corrected). Changes in FC between default mode, somatomotor, and ventral attention networks, on the one hand, and both temporal and occipital regions, on the other hand, differ significantly by age group (*p* < 0.05, corrected), and are paralleled by significant sex differences in cognitive recovery independently of age at injury (*p* < 0.05, corrected). Whereas females’ networks typically feature both significant (*p* < 0.036, corrected) and insignificant FC changes, males more often exhibit significant FC decreases between networks (e.g., between dorsal attention and limbic, visual and limbic, default-mode and somatomotor networks, *p* < 0.0001, corrected), all such changes being accompanied by significantly weaker recovery of cognitive function in males, particularly older ones (*p* < 0.05, corrected). No significant FC changes were found across 35 healthy controls aged 66–92 (20 females). Thus, male sex and older age at injury are risk factors for significant FC alterations whose patterns underlie post-traumatic cognitive deficits. This is the first study to map, systematically, how mTBI impacts FC between major human functional networks.

## Introduction

Traumatic brain injury (TBI) ranks high among public health challenges in the United States partly due to its relatively high incidence (∼0.1% annually), to its potentially debilitating sequelae, and to its relatively long recovery periods (de la Plata et al., 2008). Recuperation and clinical outcomes after TBI vary substantially by age and sex (de la Plata et al., 2008). Age at injury and biological sex have been proposed to impact clinical outcome and pathophysiology significantly (LeBlanc et al., 2006; Gölz et al., 2019), although the available evidence on sex effects is equivocal (Caplan et al., 2017).

During wakeful rest, brain activity mapped by functional magnetic resonance imaging (fMRI) is organized within resting state networks (RSNs) of functionally coupled cortical regions (Irimia and Van Horn, 2015). Yeo et al. (2011) confirmed the existence of seven canonical RSNs: the visual, somatomotor, dorsal attention, ventral attention, limbic, frontoparietal, and default mode networks [visual network (VN), somatomotor network (SMN), dorsal attention network (DAN), ventral attention network (VAN), limbic network (LN), frontoparietal network (FPN), and default mode network (DMN), respectively]. These authors illustrated how RS brain activity patterns can further be classified into 17 more granular RSNs (*N*_1_ to *N*_17_ in our notation) that arise naturally as hierarchical subdivisions of the seven canonical RSNs. Specifically, Yeo et al. (2011) implemented a sophisticated and data-driven procedure to cluster RSNs hierarchically without *a priori* assumptions. This allowed these authors to provide extensive evidence on the hierarchical clustering of the RSNs. Across individuals, both RSN classification schemes are consistent in their neuroanatomy and in how conditions like TBI affect them (Yeo et al., 2011; Badhwar et al., 2017; Kawabata et al., 2018; Irimia et al., 2020).

The TBI-related abnormalities have been reported in all seven major RSNs. For example, abnormal DMN connectivity changes can inform models of cognitive impairment and of neurodegeneration along trajectories that can lead to neurodegenerative diseases (Irimia et al., 2020). fMRI studies of TBI (Stevens et al., 2012) have identified functional correlation (FC) changes involving the FPN (Scolari et al., 2015), whose post-traumatic connectomic abnormalities can be associated with specific cognitive deficits (Hillary et al., 2011; Kasahara et al., 2011). Post-traumatic FC abnormalities also include reduced connectivity of the VN to other brain regions, even in cognitively normal and in behaviorally unimpaired individuals (Slobounov et al., 2011). TBI-related disruptions of the DAN have also been reported, particularly involving long-range and interhemispheric information exchange (Han et al., 2016). Similarly, in mTBI-affected athletes, abnormal increases in FC in the VAN have been reported (Borich et al., 2015), and related to attention deficits (Kramer et al., 2008). Similarly, the LN may undergo significant post-traumatic FC increases (Stevens et al., 2012) paralleled by decreased WM integrity (Zhu et al., 2014; Santhanam et al., 2019). Finally, altered FC between the SMN to the supplementary motor area (*N*_7_), on the one hand, and to other brain regions, on the other hand, have been reported after TBI (Han et al., 2016). Thus, cross-sectional findings on TBI-related FC abnormalities have been explored extensively.

In this study, we extend the state of the art by considering the important topic of *change* in FC after mTBI as this pertains to *cortical* RSNs. Because post-traumatic degradation of cognitive functions is often paralleled by changes in FC between RSNs (Sharp et al., 2011), quantifying such changes can provide insight into the functional substrates and anatomic profiles of neural dysfunctions associated with post-traumatic deficits. Previous fMRI studies of TBI-related RSN changes describe how participants’ FC patterns differ from those of healthy controls (HCs) (Shumskaya et al., 2012; Vakhtin et al., 2013; Rigon et al., 2016a; Vergara et al., 2017). However, hardly any study has quantified either (A) post-traumatic FC changes, (B) how age and sex modulate these changes (de Souza et al., 2020), or (C) how the changes are related to cognitive function recovery. Such understanding could help to evaluate injury-related functional alterations occurring in parallel with structural changes involving white matter (WM) degradation, which frequently underlies cognitive degradation and/or recovery. This study leverages general linear models (GLMs) of blood oxygenation level-dependent (BOLD) fMRI signals to quantify (A) mTBI-related changes in FC between RSNs within the first ∼6 months post-injury, and (B) how age at injury and sex modulate these changes. Because studying functional interactions between cortical and non-cortical networks is sufficiently complex and difficult to warrant a separate study, we here limit ourselves to the investigation of cortical networks only. To our knowledge, this is the first systematic longitudinal study to map such post-traumatic changes occurring between the acute and chronic stages of TBI. It is also the first study to map how these changes are reflected by cognitive recovery. The acute and chronic stages of TBI overlap with the approximate timeframe of the most dynamic post-traumatic brain changes pertaining to functional reorganization, degradation, and recovery. For this reason, quantifying RSN alterations between these stages is relevant to the characterization of post-traumatic neuropathophysiology underlying cognitive function dynamics after TBI.

## Materials and Methods

### Participants

This study was approved by the Institutional Review Board at the University of Southern California and was carried out in accordance with the United States Code of Federal Regulations (45 C.F.R. 46) and with the Declaration of Helsinki. Participants were recruited through community outreach (e.g., using advertisements and flyers) and/or through healthcare professionals who had referred volunteers for neurocognitive assessments and neuroimaging. All subjects who satisfied the inclusion criteria and who could provide written informed consent were invited to participate. Participants included 136 individuals with mTBI [*N* = 136; 52 females; age range: 19–79 years (y), 72 participants below age 40 years; age mean μ = 42 years, standard deviation σ = 17 years, Table 1]. To reduce recruitment bias, all individuals satisfying the study’s inclusion criteria were invited to participate and all those who did also provided written informed consent. Inclusion criteria were (a) a TBI diagnosis due to a ground-level fall involving direct head trauma; (b) a Glasgow Coma Scale score above 12 upon initial clinical evaluation (μ ± σ = 14 ± 1); (c) loss of consciousness shorter than 30 min (μ ± σ ≃ 9 ± 4 min); (d) post-traumatic amnesia of less than 24 h (μ ± σ ≃ 3.6 ± 2.1 h); (e) availability of *T*_1_-weighted MRI scans acquired both acutely and chronically after the injury, i.e., within ∼7 days and ∼6 months (μ ± σ = 5.6 ± 0.3 months), respectively, and (f) no gross TBI pathology findings on clinical MRIs. Exclusion criteria were (a) a pre-traumatic history of cognitive impairment, neurological and/or psychiatric disease, and (b) a history of psychotropic substance abuse. This retrospective study did not involve any therapeutic intervention common across all mTBI participants. A group of 35 HCs whose fMRIs had been acquired at two timepoints were selected from the Alzheimer’s Disease Neuroimaging Initiative (ADNI), whose inclusion criteria are described elsewhere (Petersen et al., 2010).

**TABLE 1:** Participants’ ages in years (y) by sex and age group, i.e., YAs and OAs.

		Age group		
Sex	Statistic	YA	OA	All
Females	Min	20	40	20
	Max	32	79	79
	μ	26	57	41
	σ	4	12	18
Males	Min	19	40	19
	Max	39	78	78
	μ	29	58	42
	σ	7	10	17
Both	Min	19	40	19
	Max	39	79	79
	μ	28	57	42
	σ	6	11	17

*Each row lists ages by sex. The three rightmost columns list age statistics by age group. The minimum (min), maximum (max), mean (μ), and standard deviation (σ) are provided. Values are rounded to the nearest integer. OA, older adult; YA, younger adult.*

### Neuroimaging and Cognitive Assessments

*T*_1_-weighted MRIs were acquired from mTBI participants using a magnetization-prepared rapid acquisition gradient echo sequence with the following parameters: repetition time = 1.95 s; echo time = 2.98 ms; inversion time = 900 ms; voxel size = 1 mm × 1 mm × 1 mm. BOLD RS fMRI time series with at least 140 volumes per session were acquired (repetition time = 3 s, echo time = 30 ms, flip angle = 80 degrees, voxel size = 3.3125 mm × 3.3125 mm × 3.3 mm, acquisition matrix = 64 × 64 × 49). All data were anonymized and de-linked. HC subjects’ *T*_1_-weighted and functional MRIs were obtained from the ADNI database^1^. ADNI was launched in 2003 as a public-private partnership, led by Principal Investigator Michael W. Weiner, MD. The primary goal of ADNI has been to test whether serial magnetic resonance imaging (MRI), positron emission tomography (PET), other biological markers, and clinical and neuropsychological assessment can be combined to measure the progression of mild cognitive impairment (MCI) and early AD. Cognition was evaluated at the two timepoints using the Brief Test of Adult Cognition by Telephone (BTACT) (Tun and Lachman, 2006; Kliegel et al., 2007). BTACT measures episodic verbal memory [immediate recall (EVMI) and delayed recall (EVMD) of words on a 15-item list], working memory span (WMS, assessed using a backward digit span task), inductive reasoning (IR, assessed using a number series completion task), processing speed (PS, measured using a backward counting task) and verbal fluency (VF, assessed using a category fluency task). The BTACT is a phone-based cognitive assessment, so administering the BTACT is logistically convenient because participants do not have to be interviewed in person. The BTACT is also reliable, as it has been extensively validated against face-to-face assessments and against longer cognitive assessments, including the 90-min in-person Boston cognitive battery (Lachman et al., 2014). *p*-values for statistical tests involving cognitive assessments were corrected for multiple comparisons using the Benjamini–Hochberg procedure with a false discover rate (FDR) of 0.05 (Hochberg and Benjamini, 1990).

### Preprocessing

*T*_1_ MRIs were segmented using FreeSurfer 6.0 (FS^2^) with default parameters (Dale et al., 1999; Fischl, 2012). FS (a) removes non-cerebral voxels, (b) transforms volumes into Talairach space, (c) normalizes signal intensities across voxels, (d) segments the gray matter, (e) tessellates the gray matter/white matter boundary, and (f) corrects surface topology. fMRI preprocessing was undertaken using the FreeSurfer Functional Analysis Stream (FS-FAST^3^) with default parameters. The stream includes frame-to-frame motion correction, frame censoring, frequency filtering for removal of scanner and physiological noise, brain masking, intensity normalization, co-registration between *T*_1_ and fMRI volumes, FS atlas surface sampling, smoothing using a kernel with a full width at half maximum of 5 mm, and volume resampling to the Montreal Neurological Institute’s regional parcellation space generated using data from 305 subjects. Nuisance timeseries due to motion, WM, and cerebrospinal fluid were regressed out and the first four volumes in each time series were removed for signal equilibration. Within a general linear model, FS-FAST was used to calculate the main effects of sex and age, as well as their interaction, on changes in FC between each of the 17 functional networks (seeds) and the rest of the cortex. Importantly, by default, FS-FAST implements a correction for multiple comparisons during the procedure for identifying statistically significant clusters. This default option for multiple comparison correction was used throughout this study.

### Functional Correlation Network Parcellation and Seeds

**FIGURE 1:**
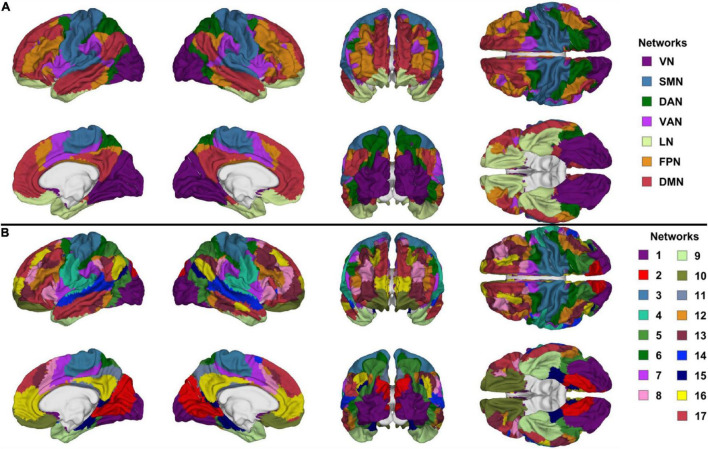
Canonical views (left, right, anterior, posterior, dorsal, ventral, and medial) of **(A)** the seven canonical RSNs and **(B)** the 17-network RSNs delineated by Yeo et al. (2011). The color scheme and nomenclature are those used in the original publication (Yeo et al., 2011). RSN, resting state network.

**FIGURE 2:**
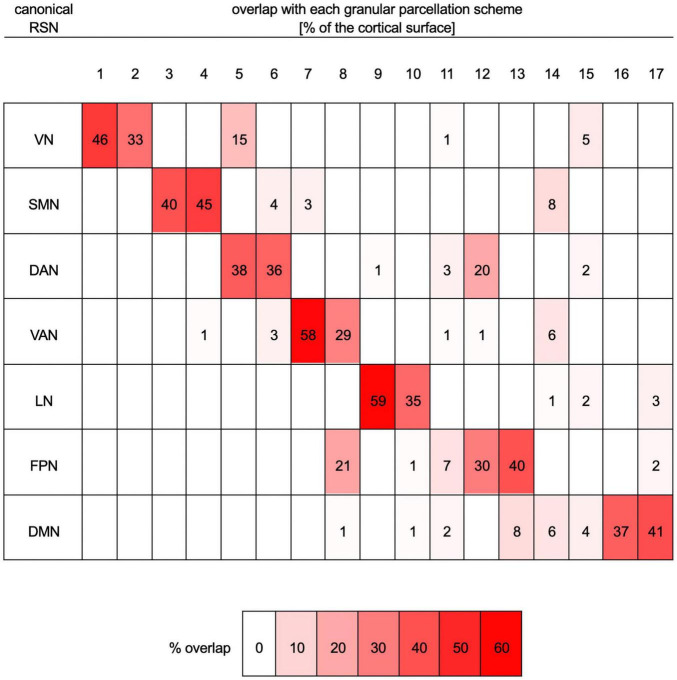
Quantitative mapping of overlaps between the parcellation scheme with seven RSNs onto the more granular parcellation scheme with 17 RSNs. Both schemes are those of Yeo et al. (2011). Each row lists data pertaining to one of the seven canonical networks (VN, SMN, DAN, VAN, LN, FPN, and DMN). Columns list data corresponding to one of the networks (*N*_1_ to *N*_17_) in the more granular 17-network cortical parcellation scheme. For each row (i.e., RSN), the table indicates the percentage of that RSN’s cortical surface area, in the seven-network scheme, that overlaps with each of the more granular networks in the 17-network scheme. For example, 46% of the VN overlaps with *N*_1_, 33% of it overlaps with *N*_2_, 15% with *N*_5_, 1% with *N*_11_, and 5% with *N*_15_. For convenience, percentages are also color-coded, where white indicates 0% overlap and bright red indicates maximum overlap (59%). DAN, dorsal attention network; DMN, default mode network; FPN, frontoparietal network; LN, limbic network; RSN, resting state network; SMN, somatomotor network; VAN, ventral attention network; VN, visual network.

Functional correlation seeds were identical to those defined by Yeo et al. (2011), who had assigned each cortical region to one of seven canonical RSNs (DMN, DAN, VAN, FPN, LN, SMN, or VN, Figure 1A). Although these networks consist of non-contiguous cortical parcels, most such parcels within each RSN exhibit significantly intercorrelated RS fMRI signals, whence their grouping into RSNs. The seven canonical RSNs can be hierarchically subdivided into 17 more granular networks *N_i_* where *i* = 1, …, 17 (Figure 1B). These 17 networks map onto the seven canonical RSNs approximately as follows: *N*_1_ and *N*_2_ map onto the VN, *N*_3_ and *N*_4_ onto the SMN, *N*_5_ and *N*_6_ onto the DAN, *N*_7_ and *N*_8_ onto the VAN, *N*_9_ and *N*_10_ onto the LN, *N*_8_, *N*_12_ and *N*_13_ onto the FPN, *N*_16_ and *N*_17_ onto the DMN (Figure 2). Because the 17-network parcellation scheme is more anatomically granular, each of its RSNs was used as a seed in the FC analysis. In the remainder of this subsection, each RSN and its functions are described to facilitate conceptual interpretation.

The VN’s primary role is in processing visual information (DeAngelis et al., 1995; Culham et al., 2001), and TBI can lead to visual deficits underpinned by VN dysfunction (Palacios et al., 2017). The VN fractionates into two subnetworks comprising central (*N*_1_) and peripheral visual (*N*_2_) areas, respectively, divided across by the calcarine fissure. *N*_1_ spans the lateral and the posterior part of the medial occipital lobe (i.e., most of the primary visual cortex, V1), whereas *N*_2_ comprises other visual areas (i.e., V2–V5). Thus, the VN contains neuronal populations attuned to detecting visual features (across both *N*_1_ and *N*_2_), object motion (*N*_2_), as well as the speed and direction of such motion (e.g., visual area V5, which is part of *N*_2_) (Dubner and Zeki, 1971; Maunsell and Van Essen, 1983). The superior temporal gyrus/auditory cortex is a part of the visuospatial attentional system which, along with the VN, is engaged in exogenous orientation to verbal instructions to perform a visual task (Mayer et al., 2004; Halgren et al., 2011).

The SMN is subdivided by separating the superior and inferior portions of the precentral gyrus (PCG) and postcentral gyrus (PoCG) by a line proceeding, in anteroposterior fashion, along the crown of the middle frontal gyrus (MFG). This produces one dorsal and one ventral network (*N*_3_ and *N*_4_, respectively), reflecting a functional subdivision of somatomotor representation areas. Insights facilitated by the 17-network parcellation help to conceptualize post-traumatic somatomotor deficits as reflecting changes in the FCs of highly specialized somatomotor representation areas. Specifically, *N*_3_ (which includes the superior dorsolateral aspects of the PCG and PoCG), is responsible for proprioceptive sensation and for the control of hands and fingers, whereas *N*_4_ (which includes the inferior aspects of the PCG and PoCG, the posterior aspect of the insula and the temporal plane), processes sensory information from the face, eyes, nose, mouth, tongue, and jaws (Grodd et al., 2001). Wernicke’s area (*N*_4_) is associated with both visual and auditory language comprehension (Binder et al., 1997). FC between the SMN (*N*_3_) and the superior frontal gyrus (SFG) is implicated in inhibitory control during no-go tasks (Nakata et al., 2009). This may partly explain why TBI patients often experience behavioral impulsivity and reduced inhibitory control (Dimoska-Di Marco et al., 2011).

The DAN is a task-positive network partly responsible for visuospatial attention (Umarova et al., 2010). The DAN contains one anterior and one posterior subnetwork (*N*_5_ and *N*_6_, respectively, Figure 2). The anterior subnetwork of the DAN (*N*_5_) comprises the anterior aspects of the inferior occipital gyrus and sulcus, the lateral occipito-temporal gyrus, the lingual part of the medial occipito-temporal gyrus, and the superior parietal lobule. The posterior subnetwork of the DAN (*N*_6_) includes posterior portions of the MFG, as well as the supramarginal gyrus, the superior parietal lobe, the precentral sulci, and the precuneus (PCun). The DAN can act in concert with the VAN, which is involved in redirecting attention to novel stimuli of behavioral relevance (Corbetta and Shulman, 2002; La et al., 2014). The fusiform gyrus (*N*_5_) is involved in high-level visual computations undertaken during face perception, object recognition, and reading (Weiner and Zilles, 2016). Worse performance on tasks related to these cognitive functions has been reported after TBI (Alnawmasi et al., 2019; Turkstra et al., 2020).

The VAN consists of two subnetworks (*N*_7_ and *N*_8_) extending across the anterior insula and parts of the frontal lobe. *N*_7_ areas are adjacent and posterior to *N*_8_ and include the SFG, portions of middle cingulate and supramarginal cortices, as well as the anterior opercular part of the inferior frontal gyrus. *N*_8_ encompasses the orbital and triangular parts of the inferior frontal gyrus, as well as the MFG, SFG, and supramarginal gyri. Specifically, the VAN specializes in detecting (A) unexpected and unattended stimuli, and (B) selecting a stimulus upon which the DAN should focus its neural processing capabilities related to sustained attention (Vossel et al., 2014). In right-handed individuals, the opercular and triangular parts of the inferior frontal gyrus together form the right-hemisphere homolog of Broca’s area (*N*_7_), which is activated by relatively complex tasks involving speech processing and word retrieval (Just et al., 1996; Drager et al., 2004; Papoutsi et al., 2009; Schremm et al., 2018). One year after TBI, patients often exhibit deficits in their voluntary control of attention, and these deficits are correlated with reductions in FC between the VAN and other regions, relative to HCs (Richard et al., 2018).

The LN consists of two subnetworks, one temporal and another frontal (*N*_9_ and *N*_10_, respectively). Deficits pertaining to the recognition of faces and facial expressions, both of which involve the LN, are more prevalent after TBI than after brain tumors or stroke by 20 and 77%, respectively (Prigatano and Pribram, 1982; Valentine et al., 2006; Knox and Douglas, 2009).

The FPN is partitioned into *N*_12_ [comprising the inferior frontal sulcus, inferior frontal gyrus, MFG, and posterior aspect of the middle temporal gyrus (MTG)] and *N*_13_ (comprising the inferior temporal sulcus, MTG, medial aspect of the SFG, superior frontal and lateral orbital sulci, as well as parts of the angular and supramarginal gyri). The cortical regions belonging to *N*_12_ and *N*_13_ are typically adjacent. Thus, *N*_12_ includes the MFG, parts of the postcentral sulci, and parts of the inferior temporal gyri (posterior aspects). *N*_13_ includes parts of the MFG, lateral orbital sulci, postcentral sulci, inferior temporal gyri, and small parts of the SFG (medial aspects). *N*_16_ overlaps with the anterior cingulate cortex, PCun, the ventral aspects of supramarginal gyri, and with part of the anterior aspect of the MTG. FC degradation in the MTG is associated with cognitive decline after injury in males (Konstantinou et al., 2018).

The DMN includes *N*_16_,which overlaps with the anterior cingulate cortex, PCun, the ventral aspects of supramarginal gyri, and part of the anterior aspect of the MTG; and *N*_17_, which contains the SFG, MTG, superior frontal sulcus, and the angular gyrus.

### Theoretical Framework for Sex and Age Differences

Our methodological approach relies on a theoretical framework for age- and sex-related differences in mTBI-related changes in FC that emerges from the functional neuroanatomy literature. This framework has not been expounded adequately before, yet its specifications are important for understanding the rationale of our approach and for the interpretations of our results. For these reasons, this theoretical framework is synthesized in what follows. Thus, age-related decline in cognitive performance is related to cortical thinning and related functional plasticity to compensate for this thinning (Greenwood, 2007). Older age has been associated with reduction in FC; for example, in older adults, Wang et al. (2010) found both a decline in frontotemporal and temporoparietal FC, as well as an increase in DMN FC during memory encoding and recognition.

The relationship between cognitive functions and FC is also modulated by sex, although a comprehensive theoretical framework explaining these differences has not been established. Male mTBI patients have been found to exhibit decreased FC in the VN compared to females, as well as increased FC across multiple networks, including an executive function-related network associated with insomnia severity (Wang et al., 2018). Functions of the PCun that involve the VN pertain to the processing of visuospatial imagery and to episodic memory retrieval (Cavanna and Trimble, 2006), both of which are often affected by TBI (Kwon and Jang, 2011; Gillis and Hampstead, 2015; Gilmore et al., 2016). PCun (*N*_2_) involvement in these functions is also modulated by sex (Butler et al., 2006; Zilles et al., 2016).

In terms of the SMN, meta-analytic findings on sex-related differences pertaining to deficits of motor function and working memory are inconclusive (Gupte et al., 2019). HC females typically achieve better inhibitory control during no-go tasks (Li et al., 2006), and males’ exhibit reduced inhibitory control manifested as willful hand stillness during motor tasks (Nakata et al., 2009). The intrinsic FC of areas recruited by motor function is affected by TBI in a sex-dependent manner (Wang et al., 2018). The supplementary motor area (*N*_3_) is likely involved in motor performance, particularly motor control (Goldberg, 1985), and TBI patients often suffer from motor deficits (Kuhtz-Buschbeck et al., 2003; Choi et al., 2012). Post-TBI, females tend to score worse than men on motor skill tests (Moen et al., 2014). Females typically demonstrate fewer somatosensory deficits following mTBI compared to males indicating sex differences in somatosensory deficit severity (Covassin et al., 2006; Bay et al., 2009). Some of these clinical observations (Irimia and Bradshaw, 2005a,b) have been attributed to females’ greater neuroprotective immune responses after TBI, which may be due to sex differences in hormone levels and endocrine function after injury (Stein, 2008). Sex-related differences in somatosensation (whose processing is localized to the PoCG) are modulated by the estrous cycle, which also predicates sex differences in neural plasticity (Alexander et al., 2018).

One of the clusters whose post-traumatic FC to the DAN (*N*_5_) changes in a sex-dependent manner overlaps, to a large extent, with the fusiform gyrus. Poorer performance during face perception, object recognition, and reading has been reported in male TBI patients (Moore et al., 2010; Wang et al., 2018). Hypoconnectivity between the VAN and occipital cortex underlies attention deficits (Farrant and Uddin, 2016), and females’ ability to sustain attention is typically greater in the chronic stages of TBI (Ratcliff et al., 2007).

Functional correlation between the LN (*N*_9_) and the lingual gyrus is directly involved in face recognition (Kanwisher et al., 1997; Rotshtein et al., 2001), which is involved in the Diagnostic Analysis of Nonverbal Accuracy task (on which males perform significantly better) (Nowicki and Carton, 1993; Babbage et al., 2018). The superior parietal lobule, which overlaps with *N*_3_ and is involved in language processing, has altered FC with the FPN after TBI in a sex-dependent manner (Segal and Petrides, 2012; Banaszkiewicz et al., 2021). After injury, this structure undergoes BOLD signal reductions, which translate into altered FC between the superior parietal lobule and other regions (Sanchez-Carrion et al., 2008).

The inferior parietal lobule (IPL) deserves individual treatment here due to its well-documented and important sexual dimorphism (Frederikse et al., 1999), which may be responsible for sex differences in visuospatial ability (Culham and Kanwisher, 2001; Zago and Tzourio-Mazoyer, 2002). Emotion perception is among the primary functions of the IPL, which overlaps with *N*_1_, *N*_5_, *N*_15_, and *N*_16_ (Engelen et al., 2015). Following injury, the ability to perceive emotions evoked by facial and by other stimuli tends to degrade (Green et al., 2004; Bornhofen and McDonald, 2008a,b,c). Furthermore, females exhibit weaker deficits in emotion perception after injury (Rigon et al., 2016b). The connectome hub localized within the IPL is among the largest in the connectome; aside from emotion processing, the IPL is also involved in language production (Barbeau et al., 2017; Southwell et al., 2017; Jiao et al., 2020), which can be affected by TBI (DePompei and Hotz, 2001; Steel et al., 2017; Koebli et al., 2020) in a sex-dependent manner. Specifically, after injury, females typically preserve their overall language abilities better than males (Covassin and Bay, 2012; Eramudugolla et al., 2014).

### Functional Correlation Analysis

Within FS-FAST, a weighted least-squares GLM implemented by the FS mri_glmfit function was used to identify cortical areas exhibiting significant FC to each seed RSN. Such areas consisted of one or more contiguous cortical surface clusters whose overall BOLD signals had statistically significant partial correlations ρ with the BOLD signal of the seed RSN, both at the acute (ρ_*A*_) and chronic (ρ_*C*_) timepoints (null hypothesis: ρ_*A*_ = ρ_*C*_). The correlation values were compared across timepoints over the entire cortical surface, rather than only across clusters of significant correlations identified by the BOLD signal analysis. The FS-FAST mri_glmfit-sim function was used to evaluate cluster-wise statistical significance using the –cwp flag. Multiple comparison correction was implemented using –3 spaces flag. We chose the *partial correlation coefficient* ρ rather than the standard *contrast effect size* as a measure of FC because ρ ranges from −1 to 1, thus making interpretations and comparisons more straightforward for our purposes. This choice does not affect the results of the study because FS-FAST takes into account both when calculating statistical significance. Here and throughout, the effect sizes discussed are those pertaining to partial correlations ρ, i.e., to FCs. Let ρ_*F*_ and ρ_*M*_ denote the partial correlation coefficients of females and males, respectively. Similarly, let ρ_*O*_ and ρ_*Y*_ denote the partial correlation coefficients of older adults (OAs) and younger adults (YAs), i.e., between adults younger vs. older than 40 years, respectively. There are three effect size types considered in this study: (A) the difference Δρ_*T*_ = ρ_*C*_−ρ_*A*_, (quantifying FC changes with time), (B) the difference Δρ_*S*_ = ρ_*F*_−ρ_*M*_ (quantifying differences between sexes), and (C) the difference Δρ_*A*_ = ρ_*O*_−ρ_*Y*_ (quantifying FC differences between age groups). Effect sizes were calculated for each subject, and all subjects’ timeseries were then concatenated for group-level statistical analysis. Significant group differences between YAs and OAs and group differences between sexes were identified within the same GLM. The age cutoff of 40 years was selected partly for convenience, as this cutoff splits our sample roughly into half. Testing null hypotheses of group contrasts allowed us to identify ρ values that differed significantly across groups. Δ*p*-values were computed clusterwise (i.e., at the cluster rather than vertex level). To facilitate reporting and tabulation, group-wise averages of partial correlations were calculated across clusters and effect sizes were reported using Cohen’s *d* multiplied by either −1 or +1, depending on whether the effect was associated with a decrease or increase in FC, respectively. Thus, for example, *d* = −0.2 indicates an FC decrease with an effect size of 0.2; analogously, *d* = +0.5 indicates an FC increase with an effect size of 0.5. To relate FCs to cognitive measures, the maximum absolute value of Δρ was first identified for each subject within each of that subject’s spatial cluster of statistical significance. In other words, for each cluster of significance that had been identified across all subjects, the peak effect size Δρ_max_ was first identified within each cluster and for each subject. Pearson’s product moment correlation coefficient between each maximum effect size Δρ_max_ and each cognitive score (EVMI, EVMD, WMS, IR, and PS) was then calculated, and its statistical significance was tested using Student’s *t*-test with *N*-2 degrees of freedom, *N* being the sample size. Figures visualizing statistical results were generated in Mathematica (Wolfram Research, Urbana-Champaign, IL, United States); cortical maps of significant statistical findings were generated in MATLAB (Mathworks, Natick, MA, United States).

### Tabulation and Reporting

To improve anatomo-functional localization, our FC analysis was implemented using the 17-network parcellation of Yeo et al. (2011) to define FC seeds, rather than the coarser seven-network (canonical) RSN parcellation. Because the 17-network parcellation is more granular, this allows one to localize age- and sex-specific effects on the cortex at relatively higher spatial resolution. Additionally, this reduces both (A) *spatial filtering effects* due to time series averaging and (B) *cancellation effects* whereby the anatomical patterns and distributions of correlation coefficients with opposite signs are lost (canceled out) when the coefficients are averaged over across relatively small cortical patches. Nevertheless, because the seven RSNs are more frequently studied, better understood, and easier to interpret, we choose to report, tabulate, and discuss results using the nomenclature of the seven-network parcellation scheme. For completeness, however, we also highlight notable anatomic and functional insights facilitated by our use of the 17-network RSN parcellation scheme. To summarize results obtained from our 17-RSN FC analysis, we leveraged the information in Figure 2, which maps the 17-RSN scheme onto the 7-RSN scheme, to map results obtained within the former onto the latter.

## Results

In this section, age- and sex-related effects on FC degradation after mTBI are reported. The text makes frequent reference to Table 2, which lists significant correlations between cognitive measures and FC changes. Because this table reports test statistics and related *p*-values, these quantities are not reported in the text; instead, the row of the table where these values can be found are reported there.

**TABLE 2:** Statistically significant correlations between FC changes involving significant cortical clusters identified through GLM and cognitive task performance.

Test	Variable	Cluster	*r*	*T*	*p*	*q*
EVMI	Sex	1	−0.148	−1.699	0.046	0.049
	Sex	4	−0.265	−3.121	0.001	0.005
	Sex	5	−0.184	−2.127	0.018	0.029
	Sex	9	−0.226	−2.635	0.005	0.014
	Sex	14	−0.173	−1.990	0.024	0.036
	Sex	16	−0.147	−1.684	0.047	0.050
	age	36	−0.181	−2.084	0.020	0.033
EVMD	Sex	9	−0.164	−1.886	0.031	0.040
	Sex	10	−0.150	−1.723	0.044	0.047
	Sex	13	−0.152	−1.747	0.041	0.046
	Sex	15	−0.181	−2.089	0.019	0.030
	Sex	25	−0.175	−2.018	0.023	0.035
WMS	Sex	1	−0.162	−1.866	0.032	0.041
	Sex	4	−0.193	−2.239	0.013	0.024
	Sex	9	−0.180	−2.082	0.020	0.034
	Sex	28	0.168	1.933	0.028	0.039
IR	Sex	10	−0.148	−1.704	0.045	0.048
	Sex	11	−0.170	−1.958	0.026	0.038
	Sex	18	0.181	2.094	0.019	0.032
PS	Sex	1	−0.265	−3.117	0.001	0.007
	Sex	3	−0.193	−2.238	0.013	0.025
	Sex	4	−0.159	−1.823	0.035	0.043
	Sex	5	−0.349	−4.225	<0.001	0.001
	Sex	6	−0.228	−2.664	0.004	0.011
	Sex	7	−0.224	−2.613	0.005	0.015
	Sex	8	−0.186	−2.148	0.017	0.027
	Sex	10	−0.309	−3.697	<0.001	0.002
	Sex	11	−0.210	−2.444	0.008	0.017
	Sex	12	−0.303	−3.605	<0.001	0.003
	Sex	13	−0.305	−3.643	<0.001	0.004
	Sex	17	−0.185	−2.132	0.017	0.028
	Sex	19	−0.274	−3.240	0.001	0.008
	Sex	20	−0.203	−2.353	0.010	0.022
	Sex	21	−0.215	−2.502	0.007	0.016
	Sex	22	−0.233	−2.717	0.004	0.012
	Sex	24	−0.236	−2.759	0.003	0.009
	Sex	26	−0.192	−2.227	0.014	0.026
	Sex	27	−0.161	−1.854	0.033	0.042
	Sex	29	−0.228	−2.664	0.004	0.013
	Sex	30	−0.205	−2.383	0.009	0.021
	Sex	31	−0.209	−2.423	0.008	0.018
	Sex	33	−0.210	−2.442	0.008	0.020
	Sex	34	−0.241	−2.819	0.003	0.010
	Sex	35	−0.201	−2.326	0.011	0.023
VF	Sex	4	−0.172	−1.984	0.025	0.037
	Sex	16	−0.159	−1.832	0.035	0.045

*Each row displays the cognitive subtest name, the biological variable (age or sex) modulating the observed effect, index of the cluster, Spearman’s correlation coefficient ρ, the t-statistic, p-value, and FDR q-value. The degrees of freedom are df = N – 2 = 134 for all tests. For example, the first row (index 1) indicates that subjects’ performance on the EVMI subtest is negatively correlated (r = −0.148) with change in FC involving cluster 1 at a significance level of p = 0.046. A negative correlation indicates that worse performance on the corresponding cognitive subtest was associated with a TBI-related increase in FC between the acute baseline and the chronic follow-up timepoints. Details descriptive of each significant cluster can be found in Table 3 (age effects) and Table 4 (sex effects).*

*All tests passed the Benjamini–Hochberg correction at FDR = 0.05.*

*df, degrees of freedom; EVMI, episodic verbal memory (immediate recall); EVMD, episodic verbal memory (delayed recall); WMS, working memory span; IR, inductive reasoning; PS, processing speed; VF, verbal fluency; GLM, general linear model; FC, functional correlation/connectivity; TBI, traumatic brain injury.*

### Age Effects

**FIGURE 3:**
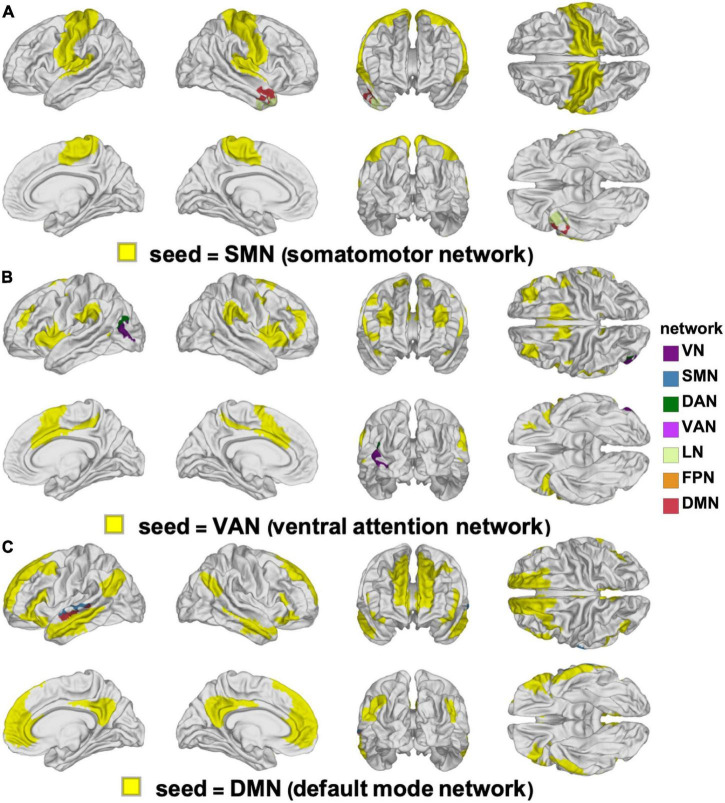
Clusters of statistically significant FC changes that differ significantly between YAs and OAs. In each panel, the seed RSN is painted in yellow and the significant clusters are painted using the color(s) of the RSN(s) with which they overlap, following the color scheme of Yeo et al. (2011), see also Figure 2. The seed networks are the SMN **(A)**, VAN **(B)**, and DMN **(C)**. No significant clusters were found for the DAN, FPN, LN, or VN. DAN, dorsal attention network; DMN, default mode network; FC, functional correlation; FPN, frontoparietal network; LN, limbic network; OA, older adult; RSN, resting state network; SMN, somatomotor network; VAN, ventral attention network; VN, visual network; YA, young adult.

**FIGURE 4:**
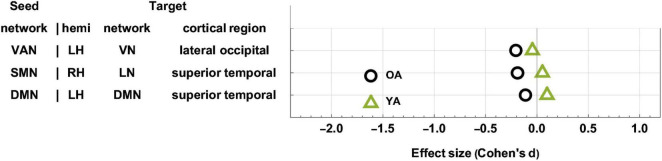
Age-related differences in FC change after TBI. OAs exhibit FC decreases across all statistically significant target clusters, whereas YAs exhibit relatively fewer significant changes across the ∼6-month follow-up period. The horizontal axis encodes effect size (Cohen’s *d*), and seed-target cluster pairs are listed in ascending order according to males’ effect size *d*. FC, functional correlation; OA, older adult; TBI, traumatic brain injury; YA, young adult.

The YAs and OAs differ significantly in how the SMN, VAN, and DMN are functionally coupled to various cortical clusters (Figure 3 and Table 3). Here and throughout, *d_Y_* and *d_O_* stand for Cohen’s *d* (the effect sizes for YAs and OAs, respectively) multiplied by the sign of the change in FC associated with *d*. We find a significant age group difference in FC changes (*d_Y_* = 0.05, *d_O_* = −0.19, Δ*d*_*A*_ = −0.24, *p* = 0.0027) between the SMN and a cortical cluster spanning a patch of lateral temporal cortex overlapping with the LN and DMN (Figure 3A). This cluster’s FC changes are significantly and negatively associated with changes in EVMI (*r* = −0.18, *t*_134_ = −2.09, *p* = 0.02, *q* = 0.03, see cluster 36 in Table 2). Another significant age difference in FC change (*d_Y_* = −0.04; *d_O_* = −0.21, Δ*d*_*A*_ = −0.25, *p* = 0.0305) is found between the VAN and a cluster spanning lateral occipital and inferior parietal regions (Figure 3B). Thirdly, a significant difference in FC change (*d_Y_* = 0.10, *d_O_* = −0.11, Δ*d*_*A*_ = −0.21, *p* = 0.0078) is found between the DMN and a cluster spanning parts of the superior temporal, middle temporal, and anterior cingulate cortices (Figure 3C). Notably, OAs exhibit relatively larger FC decreases than YAs (Figure 4). In OAs, FC decreases across all three clusters whereas, in YAs, FC is relatively unchanged for the YAs, such that |*d*_*Y*_| < |*d*_*O*_|.

**TABLE 3:** FCs quantified by effect sizes (Cohen’s *d*) for YAs and OAs.

	Effect size (*d*)				Network cluster		
Index	YA	OA	YA-OA	*p*	Seed	Target	Size [cm^2^]
1	−0.04	−0.21	0.16	0.0305	VAN ↔ LH	VN	4
					VAN ↔ LH	DAN	2
2	0.05	−0.19	0.24	0.0027	SMN ↔ RH	LN	4
					SMN ↔ RH	DMN	3
3	0.10	−0.11	0.21	0.0078	DMN ↔ LH	DMN	6
					DMN ↔ LH	SMN	

*Results are listed for each pair of cortical clusters whose FC change differs significantly by age group. Each row indexed by the integer in the first column lists results pertaining to seed-target pairs of clusters. To identify each such pair, we list the seed RSN, the hemisphere of the target cluster, and the RSN(s) that overlap(s) with the target cluster. The area of each target cluster is listed in cm^2^. The p-value associated with the statistical significance of the FC between the seed RSN and the target cluster(s) is also provided. For example, the row indexed by the numeral 1 in the table lists data pertaining to the FC between the seed network VAN (seed network) and a statistically significant cluster with an area of ∼4 cm^2^ located in the left-hemisphere portion of the VN (target network). The effect sizes of the FC change are listed; for example, for index 1, Cohen’s d for YAs is d_Y_ = −0.04 and Cohen’s d for OAs is d_O_ = −0.21, with an effect size difference d_Y_-d_O_ = 0.16.*

*DAN, dorsal attention network; FC, functional correlation; LH, left hemisphere; RH, right hemisphere; RSN, resting state network; OA, older adult; VAN, ventral attention network; VN, visual network; YA, younger adult.*

### Sex Effects

**FIGURE 5:**
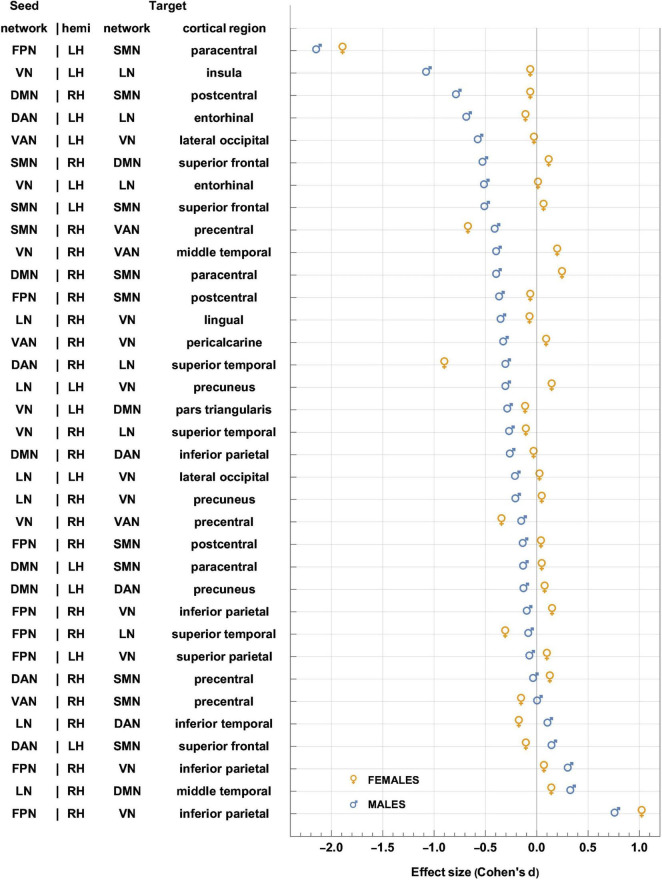
Like Figure 4, for sex-related effects. Clusters are sorted in ascending order of FC change in males. Females and males are indicated by gold ♀ and by blue ♂ symbols, respectively. Most significant findings are associated with greater FC decreases in males, whereas females’ typical significant FC changes are relatively smaller. FC, functional correlation.

Table 4 lists (A) Cohen’s *d*_*M*_ and *d*_*F*_, and (B) Cohen’s *d*_*S*_ for differences in FC change between sexes (Δρ_*S*_). These data are tabulated for each seed-target pair; the seed is an RSN, and the target is a significant cortical cluster to which the RSN is coupled. Also listed is the overlap of each target cortical cluster with various RSNs. Here and throughout, *d*_*F*_ and *d*_*M*_ stand for Cohen’s *d* (the effect size for females and males, respectively) multiplied by the sign of the respective change in FC associated with *d*. RSN-cluster pairs are sorted in ascending order of *males’* effect sizes, which range from *d*_*M*_ = −2.14 (FC decrease) to *d*_*M*_ = 0.77 (FC increase); females’ effect sizes range from *d*_*F*_ = −1.89 to *d*_*F*_ = 1.03. The clusters listed in Table 4 are also displayed on the cortical surface in Figure 5. The reason for which males—rather than females—were selected to determine the order of displays in Table 4 and Figure 5 is that males had been found to exhibit a wider range of FC changes. This made the comparison of their FCs relative to those of females easier because females’ relatively smaller average FC changes provided a pseudo-baseline, relative to which males’ FCs could be compared. To assist the reader in comparing females’ effect sizes relative to men’s, Supplementary Table 1 lists the data in Table 4 in ascending order of *females’* effect sizes. A statistical comparison of the sexes across HCs identified no statistically significant results. Thus, across all seed networks, no significant difference in FC change across timepoints was found between sexes.

**TABLE 4:** Like Table 3, for FC changes that differ significantly across males (M) and females (F).

	Cohen’s				Networks		Cluster
Index	*M*	*F*	*M*–*F*	*p*	Seed	Target	Size [cm^2^]
1	−2.14	−1.89	−0.25	0.0019	FPN ↔ LH	SMN	11
2	−1.07	−0.06	−1.01	0.0001	VN ↔ LH	LN	20
					VN ↔ LH	DMN	17
					VN ↔ LH	SMN	11
					VN ↔ LH	VAN	8
3	−0.78	−0.06	−0.72	0.0001	DMN ↔ RH	SMN	16
					DMN ↔ RH	VN	10
					DMN ↔ RH	DAN	3
4	−0.68	−0.11	−0.57	0.0001	DAN ↔ LH	LN	13
5	−0.56	−0.03	−0.54	0.0001	VAN ↔ LH	VN	32
6	−0.52	0.12	−0.64	0.0001	SMN ↔ RH	DMN	14
					SMN ↔ RH	FPN	2
7	−0.50	0.02	−0.52	0.0001	VN ↔ LH	LN	17
					VN ↔ LH	DMN	11
8	−0.50	0.07	−0.57	0.0001	SMN ↔ LH	SMN	16
					SMN ↔ LH	DMN	13
					SMN ↔ LH	DAN	4
9	−0.40	−0.67	0.27	0.0012	SMN ↔ RH	VAN	9
10	−0.39	0.20	−0.59	0.0001	VN ↔ RH	VAN	39
					VN ↔ RH	DMN	37
					VN ↔ RH	SMN	28
					VN ↔ RH	LN	21
11	−0.39	0.25	−0.63	0.0001	DMN ↔ RH	SMN	16
12	−0.36	−0.06	−0.30	0.0001	FPN ↔ RH	SMN	16
13	−0.34	−0.07	−0.28	0.0001	LN ↔ RH	VN	45
14	−0.32	0.10	−0.42	0.0001	VAN ↔ RH	VN	25
15	−0.30	−0.90	0.60	0.0001	DAN ↔ RH	LN	14
					DAN ↔ RH	DMN	9
					DAN ↔ RH	VAN	4
16	−0.30	0.15	−0.45	0.0001	LN ↔ LH	VN	16
					LN ↔ LH	DMN	6
17	−0.28	−0.11	−0.17	0.0265	VN ↔ LH	DMN	4
					VN ↔ LH	VAN	3
18	−0.26	−0.10	−0.16	0.0001	VN ↔ RH	LN	17
					VN ↔ RH	DMN	16
					VN ↔ RH	SMN	9
					VN ↔ RH	VAN	8
19	−0.25	−0.03	−0.23	0.0046	DMN ↔ RH	DAN	2
					DMN ↔ RH	VN	2
					DMN ↔ RH	DMN	2
					DMN ↔ RH	FPN	1
20	−0.20	0.03	−0.23	0.0001	LN ↔ LH	VN	72
					LN ↔ LH	DAN	10
21	−0.20	0.05	−0.25	0.0018	LN ↔ RH	VN	8
					LN ↔ RH	DMN	1
22	−0.14	−0.34	0.20	0.0110	VN ↔ RH	VAN	5
					VN ↔ RH	SMN	3
23	−0.13	0.04	−0.17	0.0241	FPN ↔ RH	SMN	8
24	−0.12	0.05	−0.17	0.0221	DMN ↔ LH	SMN	8
25	−0.12	0.08	−0.20	0.0096	DMN ↔ LH	DAN	6
					DMN ↔ LH	FPN	2
26	−0.09	0.15	−0.24	0.0027	FPN ↔ RH	VN	3
					FPN ↔ RH	DAN	2
					FPN ↔ RH	DMN	2
27	−0.07	−0.30	0.23	0.0040	FPN ↔ RH	LN	5
					FPN ↔ RH	DMN	1
28	−0.06	0.10	−0.16	0.0283	VAN ↔ LH	VN	5
29	−0.03	0.13	−0.16	0.0355	DAN ↔ RH	SMN	6
					DAN ↔ RH	VAN	1
30	0.01	−0.15	0.16	0.0285	VAN ↔ RH	SMN	4
					VAN ↔ RH	VAN	4
31	0.11	−0.17	0.28	0.0006	LN ↔ RH	DAN	5
					LN ↔ RH	VN	4
32	0.15	−0.10	0.25	0.0019	DAN ↔ LH	SMN	11
					DAN ↔ LH	VAN	1
33	0.31	0.07	0.24	0.0033	VAN ↔ RH	VN	5
					VAN ↔ RH	DMN	1
					VAN ↔ RH	DAN	1
34	0.34	0.14	0.19	0.0135	LN ↔ RH	DMN	4
					LN ↔ RH	FPN	2
35	0.77	1.03	−0.26	0.0015	FPN ↔ RH	VN	6
					FPN ↔ RH	DAN	2

*For example, the row indexed by the numeral 1 in the table lists data for FC between the FPN (seed network) and a statistically significant cluster, with an area of ∼11 cm^2^, located in the left-hemisphere portion of the SMN (target network). The effect sizes of FC changes are listed; for example, for index 1, Cohen’s d for males is d_M = −2.14 and Cohen’s d for females is d_F = −1.89, with an effect size difference d_M_-d_F_ = −0.25.*

*F, female(s); FC, functional correlation; FPN, frontoparietal network; M, male(s); SMN, somatomotor network.*

Each inset of Figure 5 displays a single seed RSN and the cortical clusters whose FCs to that RSN change in a way that is predicated significantly upon sex. Thus, as already stated, Figure 5 provides cortical displays of the results in Table 4. As Figure 5 and Table 4 indicate, males exhibit greater variability, across both source RSNs and target clusters, in the magnitudes of their FC changes. All seven RSNs exhibit sex-related differences in how their RS FCs to other areas change after injury (Figure 5 and Table 4). The magnitudes of most such changes are relatively smaller in females; for example, the effect size of the change in FC between the VN and the portion of the LN overlapping the insula is *d*_*F*_ = −0.06. By contrast, males’ FC changes are relatively larger in magnitude than females’ and most are *negative* (i.e., they involve FC *decreases*). Thus, FC between the VN and portion of the LN overlapping the insula changes significantly post-injury (*d*_*M*_ = −0.78, *p* < 0.0001). Males’ largest FC increases are between (A) the LN and the middle temporal portion of the DMN (*d*_*M*_ = 0.34, *p* < 0.0001), and (B) the FPN and the inferior parietal portion of the VN (*d*_*M*_ = 0.77, *p* < 0.0001, Figure 5).

Let Δ*d*_*S*_=*d*_*F*_−*d*_*M*_, where *d*_*F*_ and *d*_*M*_ are the effect sizes of females and males, respectively. Thus, Δ*d*_*s*_ is the difference in effect sizes between sexes. Because |*d*_*F*_| < |*d*_*M*_| in most cases, most sex differences in FC change occur in males. Thus, Figure 5 indicates that the largest sex differences observed pertain to FC changes between portions of the VN and LN that overlap with the insula, entorhinal cortex, and other temporal regions. For example, in the insula and entorhinal cortex, males’ FCs decrease more than in females by |Δ*d*_*s*_| = 1.01 and by |Δ*d*_*s*_| = 0.52, respectively (*p* < 0.0001). The second-largest, sex-related difference pertains to changes in FC between the DMN and SMN, for which |Δ*d*_*s*_| = 0.72 (DMN to the SMN portion in the postcentral gyrus) and |Δ*d*_*s*_| = 0.64 (SMN to the DMN portion in the SFG), respectively. Larger effects are found in males (*p* < 0.0001 for both clusters, Figure 5 and Table 4).

**FIGURE 6:**
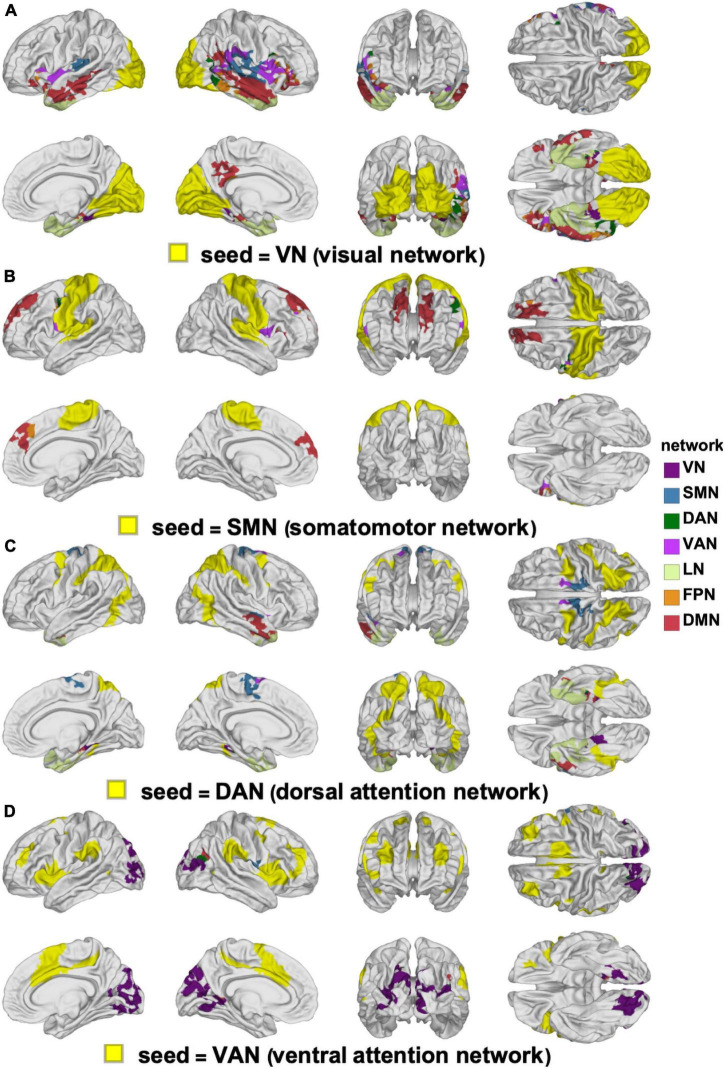
Canonical RSNs (seeds, yellow) and corresponding cortical clusters whose FC with the corresponding seed network changes significantly according to sex. The seed RSNs are the VN **(A)**, SMN **(B)**, DAN **(C)**, and VAN **(D)**. DAN, dorsal attention network; FC, functional correlation; RSN, resting state network; SMN, somatomotor network; VAN, ventral attention network; VN, visual network.

**FIGURE 7:**
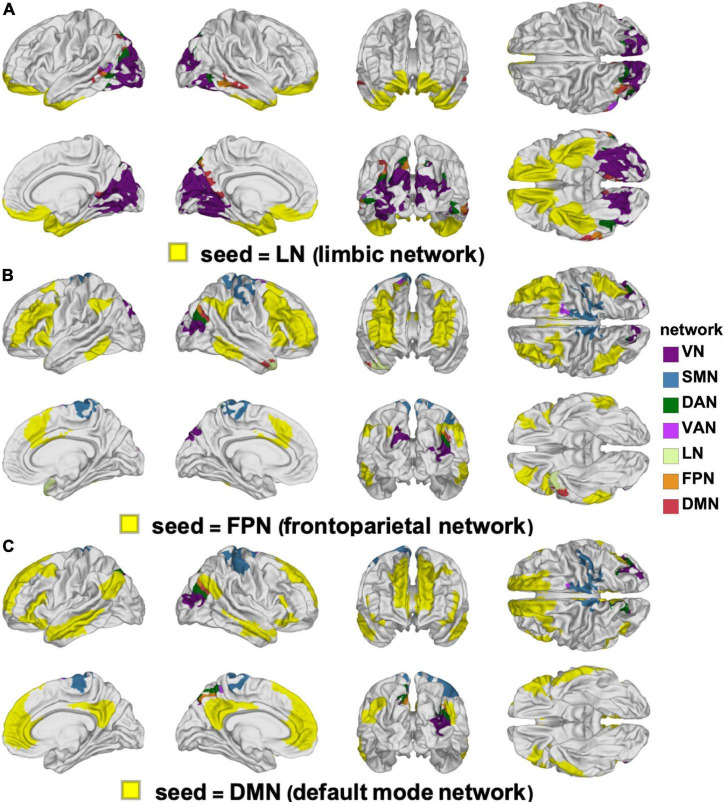
Like Figure 6, for the LN **(A)**, FPN **(B)**, and DMN **(C)**. DMN, default mode network; FPN, frontoparietal network; LN, limbic network.

Only a handful of seed-target pairs exhibit FC changes that are smaller in females (i.e., where females’ FC decreases more than males’ after mTBI), and most of these involve FCs between the DAN and other cortical regions, and between the VAN and other areas (Figures 5, 6C,D and Table 4). Examples include sex differences in FC between (A) the DAN and the superior frontal portion of the SMN (|Δ*d*_*s*_| = 0.25, *p* = 0.0019), (B) the DAN and the superior temporal portion of the LN (|Δ*d*_*s*_| = 0.60, *p* < 0.0001), and (C) the LN and the inferior temporal lobe portion of the DAN (|Δ*d*_*s*_| = 0.28, *p* = 0.0006). Other RSN-cluster pairs whose FC decreases are significantly larger in males include (A) the DAN and the entorhinal portion of the LN (|Δ*d*_*s*_| = 0.57, *p* < 0.0001), and (B) the VAN and the lateral occipital portion of the VN (|Δ*d*_*s*_| = 0.54, *p* < 0.0001) as shown in Figures 5, 6C,D and Table 4.

The clusters whose FCs to various RSNs change in ways that differ significantly by sex are mapped on the cortical surface in Figure 6 (for the VN, SMN, DAN, and VAN) and in Figure 7 (for the LN, FPN, and DMN). For each of the RSNs, there is at least one cluster whose FC to the seed RSN changes significantly in a sex-dependent manner. The pairs of regions exhibiting sex-dependent FC changes include:

(A) The VN (*N*_2_) and the MTG portion (*N*_16_) of the DMN (|Δ*d*_*s*_| = 0.59, *p* < 0.0001).
(B) The DMN (*N*_16_) and the paracentral lobule portion (*N*_3_) of the SMN (|Δ*d*_*s*_| = 0.63, *p* < 0.0001).
(C) The SMN (*N*_4_) and the PCG portion (*N*_7_) of the VAN (|Δ*d*_*s*_| = 0.27, *p* < 0.0012).
(D) The DAN (*N*_6_) and the PCG portion (*N*_3_) of the SMN (|Δ*d*_*s*_| = 0.16, *p* < 0.0355).
(E) The FPN (*N*_13_) and the superior temporal gyrus portion (*N*_9_) of the LN (|Δ*d*_*s*_| = 0.23, *p* < 0.0040).

Most clusters occur bilaterally, but do not typically overlap with a single RSN (Figures 6, 7).

### Interaction of Age and Sex

**FIGURE 8:**
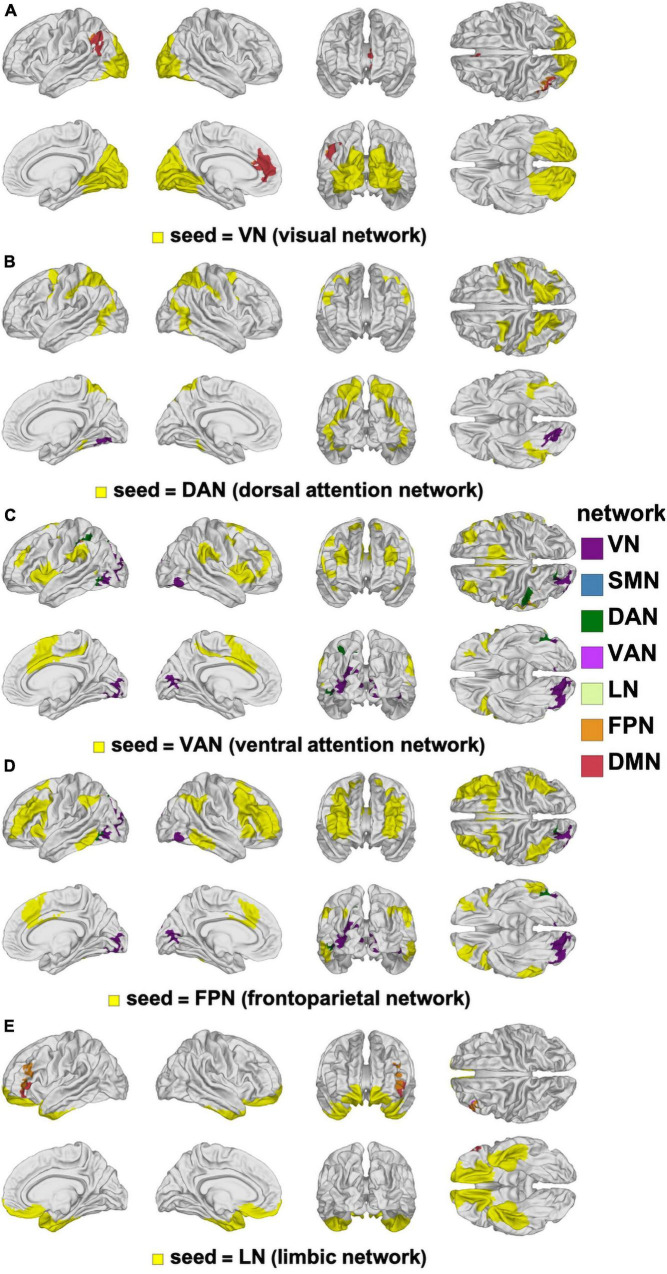
Canonical RSNs (seeds, yellow) and corresponding cortical clusters whose FC with the corresponding seed network changes significantly according to the age-by-sex interaction. The seed RSNs are the VN **(A)**, DAN **(B)**, VAN **(C)**, FPN **(D)**, and LN **(E)**. DAN, dorsal attention network; FPN, frontal parietal network; FC, functional correlation; LN, limbic network; RSN, resting state network; VAN, ventral attention network; VN, visual network.

The interaction between our main variables of sex and age were limited to small cortical clusters where significant differences were found in how FC changes depended on the sex × age interaction (Figure 8). The largest and most significant cluster is in the left supramarginal gyrus (*p* < 0.0010) and involves FC with the VN. The next most prominent clusters of interaction involve the VN seed and overlap with left rostral anterior cingulate cortex (*p* < 0.0010) and with the IPL (*p* < 0.0010).

## Discussion

In what follows, we interpret and discuss our findings by RSN, both for sex- and age-related effects. Where cognitive measures were significantly correlated with an FC change, plausible explanations, hypotheses, and postulations are provided for the change in question, along with references to similar findings in previous studies. It is important to note that these do not imply causation, given that analyses were restricted to correlations. Instead, our reasoned explanations merely convey the potential neuropsychological basis for sex- or age-related changes in FC following mTBI.

### Age Effects

Three FC clusters were found to be significantly different between OAs and YAs with mTBI (one in the left lateral part of the occipital lobe, and one in each hemisphere’s superior temporal lobe, Figures 3, 4). These clusters feature FC decreases that are significantly greater in OAs (Table 3), potentially reflecting their poorer outcomes, whether neurological, cognitive, or affective (Mosenthal et al., 2002; Mushkudiani et al., 2007; Senathi-Raja et al., 2010). In particular, the lateral occipital gyrus is recruited by visual memory, a cognitive process that is more severely affected in OAs than in YAs (Larsson and Heeger, 2006; Senathi-Raja et al., 2010; Nagy et al., 2012; Byom et al., 2019). The lateral occipital gyrus also exhibits reduced FC to the VAN after mTBI, a phenomenon we found to be associated with poorer immediate recall (EVMI) in OAs (see Figures 3, 4, and row 1 in Table 3). This abnormal connectivity between the lateral occipital gyrus and the VAN may underlie impairments of visual memory, since visual mnemonic aids supported by the lateral occipital gyrus can be employed as encoding strategies on verbal memory tasks to improve recall (Unsworth et al., 2019). Two other clusters were identified here whose FC changes differ significantly by age group, and both overlap substantially with the superior temporal gyri. These bilateral clusters exhibit FCs to the SMN and DMN, respectively, whose post-traumatic FC decreases are significantly larger in older adults (Table 3). The superior temporal lobe is involved in language processing and in emotion perception, both of which frequently degrade after TBI (Green et al., 2004; Bornhofen and McDonald, 2008a,c). Thus, our findings may provide insight on how age modulates the extent of cognitive impairments after mTBI.

### Sex Effects

**FIGURE 9:**
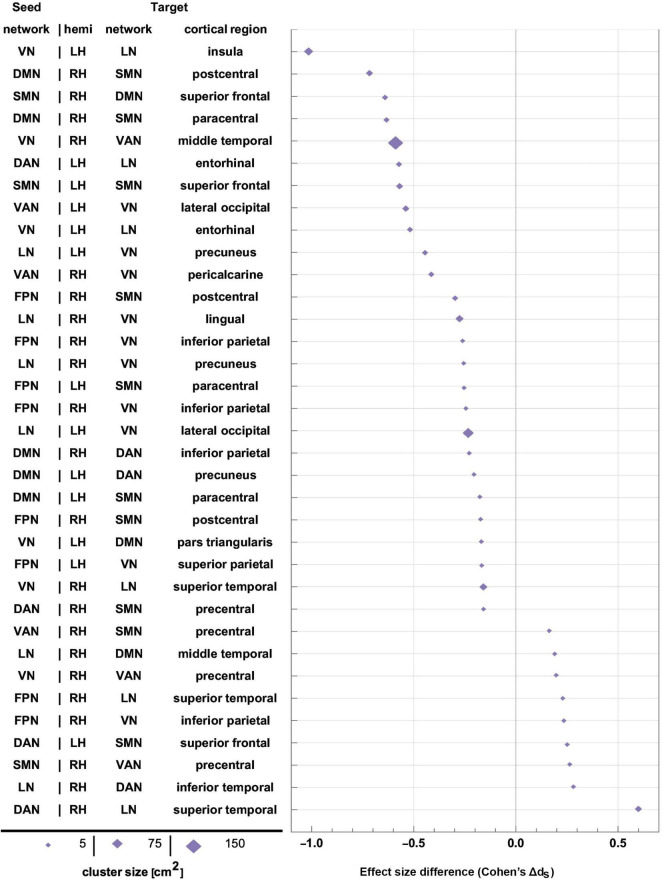
Effect sizes of FC changes predicated significantly upon sex. Pairs of RSNs (seeds) and corresponding statistically significant clusters (targets) are listed according to sex differences in effect size (Cohen’s *d*_*S*_=*d*_*M*_-*d*_*F*_, where *d_M* and *d_F* are Cohen’s *d* values for males and females, respectively). Values of *d_S* are plotted on the horizontal axis, sorted in ascending order. Marker size is proportional to cortical cluster size. FC, functional correlation; RSN, resting state network.

The sex effects identified here involve all seven canonical RSNs. Typically, males demonstrated FC decreases compared to females, who either showed smaller decreases, no difference, or increases (Figures 5, 9, and Table 4). We also observed that the typical *magnitude* of FC changes in males is larger than in females (Figure 9 and Supplementary Figure 1). For these reasons, we highlight male sex as an independent risk factor for functional network degradation after mTBI, in agreement with previous work (Gupte et al., 2019; Robles et al., 2021). Tables 5–11 provide systematic interpretations for each result, cluster by cluster. Typically, across all seed networks, we found evidence for greater FC decreases in males; in many cases, decreases were correlated with poorer cognitive performance.

**TABLE 5:** Functional neuroanatomy localizations and putative interpretation of sex-related findings pertaining to the visual network (VN).

Target	Potential interpretations of sex-related findings
MTG	mTBI involves FC decreases between the VN and the MTG in males but increases in females (Table 4). The strength of the FC between the VN (*N*_2_) and the MTG has been associated with the rationality of decision-making in a visuospatial reach-to-grasp task (Jastorff et al., 2011). Thus, our findings suggest the testable hypothesis that, all other things being equal, males’ decision-making ability in such tasks is more severely compromised, on average, than that of females. The MTG subserves a range of executive functions including language and semantic memory and is part of the downstream component of the visual association cortex (Mesulam, 1998). Accordingly, the larger FC decline between the VN and the MTG was correlated with worse performance for IR, as well as EVMD and PS (*r* = −0.15, *t*_134_ = −1.723, *p* = 0.04, *q* = 0.05, see cluster 10 in Table 2). This finding is widely supported by previous literature, including the fact that FC changes in the VN (*N*_2_) modulate males’ post-traumatic decline in non-verbal abstract reasoning (Hawes et al., 2012; Livny et al., 2017).
Insula	After mTBI, females typically perform better on tests of abstract reasoning, memory, and executive function (Niemeier et al., 2007; Saban et al., 2011). Thus, the FC changes (i.e., FC decreases in males vs. no FC changes or FC increases in females) reported here between the VN (*N*_2_) and the insula (*N*_4_) agree with prior reports of males’ post-traumatic decline in abstract reasoning abilities (Livny et al., 2017).
Wernicke’s area	After TBI, males often perform worse on language comprehension tasks that quantify reading speed and the ability to identify letters (LeBlanc et al., 2021; Pei and O’Brien, 2021). In our study, sex modulates FC decreases between the VN (*N*_1_) and Wernicke’s area (*N*_4_), which may reflect a neurophysiological mechanism that explains higher risk of chronic FC changes that affect language function and reading ability in male mTBI patients. In support of this, reductions in FC between the VN and the right superior temporal homolog of Wernicke’s area were correlated with IR (*r* = 0.18, *t*_134_ = 2.09, *p* = 0.02, *q* = 0.03, see cluster 18 in Table 2), i.e., the ability to solve abstract visuospatial and numerical problems. Wernicke’s area is associated with both visual and auditory language comprehension (Binder et al., 1997).
PCG	Greater changes in FC between the VN and the PCG, thought to have a role in visual to auditory encoding (Kaestner et al., 2021), are associated with slower PS during a backward counting task. Larger FC changes between the VN and the entorhinal cortex of the medial temporal lobe [involved in arithmetic processing (Umbach et al., 2019) are also associated with slower PS]. Interestingly, areas of both the medial temporal lobe and the VN are employed by the visuospatial sketchpad, which subserves tasks such as backward counting (Hubber et al., 2014). This was used here to measure PS. Our findings provide testable hypotheses to understand the mechanisms behind males’ decreased FC of the VN that underlie traumatic neuropathophysiology within these cognitive areas (Moore et al., 2010; Wang et al., 2018).

*The VN (seed) has FC with each listed cluster (target), and the FC in question differs significantly by sex. Putative neuroanatomical and cognitive interpretations of findings are provided in order by network index, according to the 17-network parcellation scheme.*

**TABLE 6:** Like Table 5, for the SMN.

Target	Potential interpretations of sex-related findings
PCG	Our analysis identifies two frontal clusters (indices 6 and 11 in Table 4) whose FC to the SMN decreases more significantly in males (Figures 5, 6B). These clusters include the left and right SFGs (*N*_17_), left PCG (*N*_6_), and right PCG (*N*_8_). Our findings highlight changes in FC between *N*_3_ and the PCG (motor function), as well as between *N*_4_ and the SFG (working memory). Aside from hosting the primary motor cortex, the right PCG also participates in oculomotor mapping, a function frequently impaired following TBI (Kraus et al., 2007; Hunt et al., 2016). Oculomotor function is primarily facilitated by the ventral portion of the SMN, which is located within *N*_4_ (Penfield and Boldrey, 1937). Thus, our finding of FC decrease, in both sexes, between the SMN (*N*_4_) and the left PCG may reflect how FC abnormalities between these regions reflects deficits of oculomotion within the broader context of somatomotor deficits (Iacoboni et al., 1997). Greater FC change over time between the SMN and the right PCG is also related to poorer memory abilities (including EMVI, EVMD, and WMS, see indices 9 in Table 3, respectively), which may reflect an impaired ability to encode and to map new visual and auditory information in a retrievable manner for working memory processes.
SFG	Motor function and working memory impairments have relatively high incidence even after mild injury [7 and 18%, respectively (Gagnon et al., 1998; Covassin et al., 2006; Bay et al., 2009; Choi et al., 2012)]. In agreement with lesion studies (du Boisgueheneuc et al., 2006), greater FC changes are observed between *N*_4_ and the SFG in males. It is not clear that our findings reflect degradation of neural function mediating movement and working memory, although this is plausible (Hsu et al., 2015, 2016). Facial somatosensory regions, such as those that overlap with the SFG, aid in encoding verbal information that is mentally replayed and recoded into conceptual information (Linden, 2007). Accordingly, we found that greater FC degradation was associated with lower EVMI and hypothesize that hindered integration of executive functioning and language processing with facial movement may impact verbal recall. This abnormal FC was correlated with PS (indices 6 and 8 of Table 3), indicating that impeded top-down executive control, coordinated by the bilateral SFG as part of both the SMN and DMN, may impact processing speed (Kochunov et al., 2010; Karbasforoushan et al., 2015). Our finding of males’ reduced FC between the SMN (*N*_3_) and the SFG is congruent with reported sex differences in inhibitory control manifested as willful hand stillness during motor tasks (Nakata et al., 2009).
Inferior frontal gyrus	The opercular and triangular parts of the inferior frontal gyrus (the right-hemisphere homolog of Broca’s area) form a cluster (index 23 in Table 4) whose FC to the SMN decreases significantly in both sexes (Figures 5, 7B and Table 4). In this study, participants of either sex experience decreased FC between the SMN and Broca’s area, suggesting the hypothesis that the cluster in question is recruited acutely after TBI to compensate deficits.

**TABLE 7:** Like Table 5, for the DAN.

Target	Potential interpretations of sex-related findings
Superior temporal gyri	FC between the DAN (*N*_5_) and the superior temporal gyri (*N*_4_) decreases in both males and females with mTBI (Figures 5, 6C and Table 4). Abnormal FC of these areas has been implicated in attentive listening (Braga et al., 2016), and future studies should investigate whether our finding reflects the neurophysiological substrates of post-traumatic impairments in attentive listening and in other cognitive functions involving attention (Turkstra et al., 2004). Auditory short-term memory has been shown to be affected by lesions to the superior temporal gyrus (Takayama et al., 2004). Accordingly, greater FC change between the DAN and superior temporal gyri was correlated with poorer EVMD scores (*r* = −0.18, *t*_134_ = −2.09, *p* = 0.02, *q* = 0.03, see cluster 15 in Table 2), suggesting disruption in active listening that may lead to poorer delayed recall for verbal working memory.
Supplementary motor area	Our findings agree with previous reports of increased FC between the supplementary motor area and the DAN (*N*_6_) in male patients (Wang et al., 2018), such that motor deficits observed after mTBI may reflect post-traumatic functional connectome changes between the DAN (*N*_6_) and the supplementary motor area.
PCG	Greater FC changes between the DAN and the PCG, which includes primary motor cortex, is also associated with slower PS (*r* = −0.23, *t*_134_ = −2.66, *p* = 0.004, *q* = 0.013, see cluster 29 in Table 2). This may reflect a potential mechanism whereby disruptions between attentional control and motor production can impact response speed after mTBI (Hong et al., 2015).
Entorhinal cortex	Weaker FC between entorhinal cortex and the DAN is associated with lower cognitive scores, including EVMI, WMS, PS, and VF (cluster 4 in Table 2). The entorhinal cortex is a network gateway between the hippocampus and a range of association areas that overlap with the DAN, subserving memory (Takehara-Nishiuchi, 2014), and impulse control (Matuskova et al., 2021).

**TABLE 8:** Like Table 5, for the VAN.

Target	Potential interpretations of sex-related findings
LOC	This study finds larger post-traumatic FC decreases in males between the VAN (*N*_7_) and the lateral occipital cortex (LOC), which play a major role in regulating attention (Tallon-Baudry et al., 2005). Following mTBI, males usually exhibit more severe attention deficits (Ratcliff et al., 2007). Males in our study exhibit decreases in FC between the VAN (*N*_7_) and the LOC, whereas females’ FC changes relatively little (Figures 5, 6D and Table 4). Larger FC changes are also associated with poorer EVMI and PS (index 5 in Table 2), in agreement with findings that recall and backward counting tasks both require sustained attention for accurate responses (Bunting et al., 2008). This may imply that post-traumatic changes in FC between the latter two regions reflect sex differences in attention recovery. Our findings of post-traumatic FC decreases involving the VAN agree with those of studies reporting attention difficulties and FC abnormalities in this network (Guell et al., 2020; Mallas et al., 2021).
Pericalcarine cortex	The pericalcarine cortex is found to have sex-dependent FC changes after mTBI (Figures 5, 6D and Table 4). This region aids in the modulation of attention independently of other anatomic structures within the VAN (*N*_7_) (Siegel et al., 2008). Males, but not females, exhibit reduced FC between the pericalcarine portion of the VAN (*N*_7_) and other cortical regions. Greater FC changes between the VAN and pericalcarine cortex are also associated with poorer EVMI (index 14 in Table 2). Jiang et al. (2016) found that thicker pericalcarine cortex is associated with better attention during cognitive tasks. Thus, post-traumatic attentional problems, especially common in males, may be modulated by FC between the VAN and pericalcarine cortex.

**TABLE 9:** Like Table 5, for the LN.

Target	Potential interpretations of sex-related findings
Inferior temporal gyrus	For recognition of faces and facial expressions, the brain recruits FC between the LN and inferior temporal areas, such as the lingual (LinG) and fusiform gyri (Kanwisher et al., 1997; Rotshtein et al., 2001). This FC is frequently impaired by frontotemporal TBI (George et al., 1999; Furl et al., 2011). According to our findings, FC involving both *N*_9_ and *N*_10_ is affected differentially by sex; thus, on average, males had significant FC decreases—females had increases—between the LN and the fusiform face area (Figures 4, 6A and Table 4). Furthermore, greater FC changes between the LN and inferior temporal gyrus were correlated with PS. The inferior temporal gyrus is involved in orthographic processing (Rajalingham et al., 2020), and disconnection between it and the LN may hinder mental imagery supporting tasks such as backward counting.
Lingual gyrus	The lingual gyrus (*N*_16_) is involved in episodic memory, emotional stimulus processing, and consciousness (Utevsky et al., 2014; Liu et al., 2017). This region often exhibits decreased FC in clinically depressed patients with schizophrenia or Alzheimer’s disease (Utevsky et al., 2014; Liu et al., 2017; Forlim et al., 2020). The lingual gyrus (*N*_2_, a VN subnetwork) is involved in the maintenance of consciousness, in visual processing, and in logic and arithmetic operations (Dehaene and Dehaene-Lambertz, 2009; Rosenberg-Lee et al., 2011). TBI frequently affects reading and arithmetic abilities, especially in children (Kinsella et al., 1995; Ewing-Cobbs et al., 1998, 2004; Catroppa and Anderson, 1999). The lingual gyrus and PCun belong either to the VN and/or DMN, and are thought to mediate emotional processing (Figures 5, 7A and Table 4). The clusters identified here whose FCs to the LN (*N*_9_ and *N*_10_) degrade in a sex-dependent fashion extend bilaterally across the PCun, lingual gyrus, and LOC, including the lateral occipital gyri (Figure 7A). Our findings suggest that males typically experience significant decreases in FC between the LN and all three clusters relative to females (Table 4). Large studies with sample sizes ranging from *N* = 774 to *N* = 12,605 (Iverson et al., 2011, 2013; Singh et al., 2018) unequivocally identify women as being at higher risk for depressive symptoms after mTBI (Bay et al., 2009; Gupte et al., 2019). Thus, the available evidence highlights the need for further investigation on the relationship between TBI, emotional regulation and clinical depression.
PCun	The PCun (*N*_16_) is involved in episodic memory, emotional stimulus processing, and consciousness (Utevsky et al., 2014; Liu et al., 2017), exhibiting decreased FC in clinically depressed patients with schizophrenia or Alzheimer’s disease (Utevsky et al., 2014; Liu et al., 2017; Forlim et al., 2020). Larger FC changes between the LN and the bilateral PCun are associated with EMVI, VF, and PS (indices 16 and 21 in Table 2). The PCun is recruited in several cognitive processes, including verbal episodic memory retrieval (Cavanna and Trimble, 2006), such that our findings are congruent with the known functions of this structure.
LOC	Significant FC decreases between the LN and the LOC (*N*_1_), known to participate in facial emotion recognition (Nagy et al., 2012), are found in males (Figures 5, 7A and Table 4). This suggests the testable hypothesis that decreased FC between the LN and the occipital regions of the VN underlies, at least partially, males’ poorer performance in tasks of facial emotion recognition (Rigon et al., 2016b). Greater FC change between these regions is correlated with slower PS (index 20 in Table 2), perhaps indicating that disconnection between visual and linguistic neural networks impedes the speed of cognitive processing.

**TABLE 10:** Like Table 5, for the FPN.

Target	Potential interpretations of sex-related findings
IPL	This study finds larger FC decreases in males between the FPN and the IPL, suggesting connectomic underpinnings for our observed sex discrepancies in affect deficits after TBI. Larger FC decreases between the IPL and the FPN in males suggests that sex differences in language abilities after injury can be underlain by the functional brain network changes reported here. The greater changes in FC between (A) the FPN and (B) both the superior temporal gyri and the IPL correlated with slower PS (indices 26, 27, 33, and 35 in Table 2). These gyri are implicated in auditory comprehension (Binder et al., 1997) and language production (Geranmayeh et al., 2012) respectively, suggesting a TBI-related, sex-specific hindrance of the FPN’s spatial working memory abilities and linguistic production essential for a backward counting task.
Superior parietal lobule	We observe sex-modulated FC decreases between the FPN and the superior parietal lobule, which is involved in language processing (Segal and Petrides, 2012; Banaszkiewicz et al., 2021). These decreases are also correlated with WMS. After injury, females typically preserve their verbal memory abilities better than males (Covassin and Bay, 2012; Eramudugolla et al., 2014). Our results show male FC decreases between the superior parietal lobule portion immediately posterior to the PCG and the FPN; on the other hand, our results identify FC increases in females (Figures 5, 7B and Table 4). We suggest that these post-traumatic FC changes may partly underlie sex differences in verbal memory.

**TABLE 11:** Like Table 5, for the DMN.

Target	Potential interpretations of sex-related findings
IPL	All cortical regions spanned by the DMN are found to experience significantly greater FC decreases in males (Figures 5, 7C and Table 4). One such finding of note involves the sex-dependent FC change between the DMN (*N*_16_), and the IPL. The anterior cingulate cortex facilitates somatosensory awareness (Khalsa et al., 2009), whose height differs by sex (Lane et al., 1998). The IPL, whose FC to the DMN (*N*_16_) decreases more in males (Figures 5, 7C and Table 4), is involved in the processing of visual and somatosensory information (Zago and Tzourio-Mazoyer, 2002; Akatsuka et al., 2008). Because females typically demonstrate fewer somatosensory deficits following mTBI compared to males’, our findings are compatible with clinical observations of sex differences in somatosensory deficit severity (Covassin et al., 2006; Bay et al., 2009). Our findings involving the IPL highlight how sex differences in the post-traumatic degradation of brain function could extend beyond anatomic features to include recovery patterns. Furthermore, this change is associated with slower PS (index 19 in Table 2). As both the DMN and the IPL are involved in a broad range of cognitive tasks, weaker FC between the DMN and the IPL may hinder the role each play in efficient generalized internal mental state processes (Broyd et al., 2009).
PoCG	We observe sex-modulated decreases in FC between the PoCG and the DMN. The PoCG is involved in somatosensory perception and nociperception, both of which differ by sex in the general population (Mercadillo et al., 2011; DiGuiseppi and Tadi, 2020). This alteration is also associated with PS (index 3 in Table 2), suggesting that lower FPN connectivity to the PoCG and paracentral lobule, subserved by the superior longitudinal fasciculi, is important for efficient PS (Turken et al., 2008). Our finding suggests the testable hypothesis according to which females’ faster/greater post-traumatic recovery of FC pertaining to somatosensation is mediated by endocrine factors.
PCun	Greater sex-modulated FC changes are observed between the DMN and the PCun, which is essential for episodic memory retrieval (Cavanna and Trimble, 2006). Accordingly, greater FC changes are correlated with poorer EMVD, as expected (index 25 in Table 2).

The largest sex differences observed pertain to FC changes between portions of the VN and LN that overlap with the insula, entorhinal cortex, and with other temporal regions. Interestingly, these regions have been highlighted extensively by FC studies of AD and are associated with memory deficits (e.g., Killiany et al., 2000; Jack et al., 2002; Chen et al., 2016). TBI patients are at higher risk of AD, especially after repeated brain injuries (Faden and Loane, 2015). Irimia et al. (2020) found overlap in the DMNs of TBI and AD patients that include medial and lateral areas of the temporal lobe. These authors found extensive commonalities in DMN FC between AD and TBI patients, and between both patient groups and HCs. The extent of AD-like FC degradation within the DMNs of geriatric TBI patients were predicted based on their acute cognitive assessment scores, with specificity and sensitivity comparable to AD blood and imaging biomarkers. These findings may be related to the fact that the thickness of entorhinal cortex can predict cognitive decline with age and cognitive impairment severity in AD (Kordower et al., 2001; Velayudhan et al., 2013; Zhou et al., 2016). This is only one example of the complex relationship between mTBI-related changes in structural and functional connectomics, whose exploration is outside the scope of this study. Our results do support previous research that highlights similarities between mTBI and AD in terms of their shared FC degradation trajectories, and the reader is referred to our previous study on this topic for further details (Irimia et al., 2020).

In HCs, we found no significant sex differences in FC changes between baseline and follow-up. Prior HC studies found relatively small changes in FC—and even no significant changes—across follow-up periods of 2–3 years (Odish et al., 2015; Ousdal et al., 2020). The absence of significant findings in our analysis is consistent with these prior studies and suggests strongly that our findings are related to mTBI effects rather than to typical aging across the timespan between imaging sessions. Presumably, any changes in HCs across 6 months, if any, are likely to be very small relative to those identified here in the mTBI group.

### Interaction of Age and Sex

Although present, the interactions between the main effects of age and sex were limited to small clusters located primarily in the left hemisphere (IPL, supramarginal gyrus, and anterior cingulate cortex). Interpretation of the age × sex interaction within these regions is not straightforward due to their small spatial extent and effect size. Furthermore, the available functional neuroanatomy literature does not provide the spatial specificity required to formulate cautious interpretations of our findings at a level of spatial localization similar to that of the interaction clusters identified here.

### Limitations

In this study, FC analysis results were mapped from the 17 RSN-scheme onto the seven canonical RSN-scheme. The advantages of this technique included (A) improved anatomo-functional localization, and (B) greater ability to summarize results across RSNs, since the 7-RSN scheme has fewer networks. Nevertheless, we did not use the same scheme for both the analysis and the summary, which may have caused relatively small inaccuracies when mapping results from one scheme onto the other. One additional limitation of this study is that, for logistical and methodological reasons, we did not study changes in FC between cortical regions and the subcortex. Future studies, however, should investigate these changes. Because fMRI can only resolve low-frequency BOLD signals, FCs involving high-frequency brain activity cannot be captured well using this technique. Alternative approaches like electro- and magnetoencephalography should be used to confirm and extend our studies further (Irimia et al., 2013a,b).

To find age-related effects, we split our cohort into two groups, each consisting of adults older or younger than 40, respectively. This threshold was convenient partly because it split our sample into approximately equal groups, thus resulting in a relatively balanced statistical design. However, because age is a continuous (rather than discrete) variable, the threshold in question is relatively arbitrary and other cutoffs may lead to different results (Irimia et al., 2015). One alternative to our approach could involve studying FC changes as a function of age at injury; nevertheless, due to the complexity of our findings, we deemed our current strategy to be preferable. Furthermore, although we did not interpret the statistical interaction between age and sex, this interaction was included in the GLM and its effect was regressed from our analysis before calculating effect sizes and FCs. Future studies should explore both the age-by-sex interaction and how FCs change as a function of age at injury.

Our sample focused on mTBI, which may limit the generalizability of our findings to moderate or severe TBI patients. Whereas explicit analysis of these severities is required, we speculate that the effects of older age and male sex on FC changes and cognitive outcomes would be exacerbated in a moderate-to-severe TBI group (Konstantinou et al., 2018). Bonnelle et al. (2011) found increased FC of the DMN in moderate-to-severe TBI patients, which correlated with deficits in sustained attention, indicating that higher TBI severity may lead to greater FC changes and poorer cognitive outcome. Finally, our study is observational rather than interventional; development and testing of interventions for mTBI-related impairments is beyond the scope of our work.

## Conclusion

This study is the first to systematically investigate of FC changes in mTBI, with a focus on how RSN connectivity is modulated by age and sex. Using GLMs, we identified FCs between canonical RSNs that undergo significant changes between the acute and chronic stages of mTBI. Group comparisons reveal that older age at injury and male sex are risk factors for post-injury functional brain network degradation. In comparison to HCs, this study involves a longitudinal design that explores, in a systematic way, FC changes across all seven canonical RSNs of the human brain. We also detail potential neurophysiological substrates—as conceptualized in terms of RSN connectivity changes—that may underlie the cognitive deficits in memory, processing speed, verbal fluency, and abstract reasoning typically observed post-mTBI, especially in males. Importantly, FC changes involving the VAN and DAN may explain post-injury impairments of visual memory and attention, respectively. FPN changes may relate to the underpinnings of language processing deficits commonly observed in mTBI patients, and post-traumatic FC changes involving the DMN may be responsible for degradations in processing speed and memory. FC changes pertaining to the SMN can help to explain the functional connectomic bases of mTBI patients’ abnormal PS and the VN- and LN-related FC alterations aid in explaining the deficits of abstract reasoning and language processing abilities frequently documented after mTBI. These results constitute substantial progress in scientific understanding of the relationships between TBI, age, sex, and cognitive outcome as reflected by FC and its changes post-injury. Future research that examines the hypotheses proposed here will provide additional insight into the pathophysiological mechanisms underlying functional connectome changes after mTBI.

## Alzheimer’s Disease Neuroimaging Initiative

Data collection and sharing for this project was funded by the Alzheimer’s Disease Neuroimaging Initiative (ADNI) (National Institutes of Health Grant U01 AG024904) and DOD ADNI (Department of Defense award number W81XWH-12-2-0012). ADNI was funded by the National Institute on Aging, the National Institute of Biomedical Imaging and Bioengineering, and through generous contributions from the following: AbbVie, Alzheimer’s Association; Alzheimer’s Drug Discovery Foundation; Araclon Biotech; BioClinica, Inc.; Biogen; Bristol-Myers Squibb Company; CereSpir, Inc.; Cogstate; Eisai Inc.; Elan Pharmaceuticals, Inc.; Eli Lilly and Company; EuroImmun; F. Hoffmann-La Roche Ltd and its affiliated company Genentech, Inc.; Fujirebio; GE Healthcare; IXICO Ltd.; Janssen Alzheimer Immunotherapy Research & Development, LLC.; Johnson & Johnson Pharmaceutical Research & Development LLC.; Lumosity; Lundbeck; Merck & Co., Inc.; Meso Scale Diagnostics, LLC.; NeuroRx Research; Neurotrack Technologies; Novartis Pharmaceuticals Corporation; Pfizer Inc.; Piramal Imaging; Servier; Takeda Pharmaceutical Company; and Transition Therapeutics. The Canadian Institutes of Health Research is providing funds to support ADNI clinical sites in Canada. Private sector contributions are facilitated by the Foundation for the National Institutes of Health (www.fnih.org). The grantee organization is the Northern California Institute for Research and Education, and the study is coordinated by the Alzheimer’s Therapeutic Research Institute at the University of Southern California. ADNI data are disseminated by the Laboratory for Neuro Imaging at the University of Southern California.

## Data Availability Statement

MRI data acquired from HC and AD participants are publicly available from the ADNI database (http://adni.loni.usc.edu). For TBI participants, primary data generated during and/or analyzed during the current study are available subject to a data transfer agreement. At the request of some participants, their written permission is additionally required in some cases.

## Ethics Statement

The studies involving human participants were reviewed and approved by Institutional Review Board at the University of Southern California. The patients/participants provided their written informed consent to participate in this study.

## Author Contributions

AA: methodology, software, formal analysis, investigation, writing – original draft, writing – review and editing, and visualization. AM: methodology, software, formal analysis, investigation, data curation, writing – original draft, and writing – review and editing. PI: investigation, writing – original draft, and review and editing. MH: investigation and writing – review and editing. TF: investigation. AI: conceptualization, funding acquisition, project administration, supervision, writing – review and editing, and resources. All authors approved the final version of the manuscript for publication.

## Conflict of Interest

The authors declare that the research was conducted in the absence of any commercial or financial relationships that could be construed as a potential conflict of interest.

## Publisher’s Note

All claims expressed in this article are solely those of the authors and do not necessarily represent those of their affiliated organizations, or those of the publisher, the editors and the reviewers. Any product that may be evaluated in this article, or claim that may be made by its manufacturer, is not guaranteed or endorsed by the publisher.

## Footnotes

1
adni.loni.usc.edu

2
https://surfer.nmr.mgh.harvard.edu

3
https://surfer.nmr.mgh.harvard.edu/fswiki/FsFast

## Abbreviations

ADNI: Alzheimer’s disease neuroimaging initiative
BOLD: blood oxygenation level-dependent
BTACT: brief test of adult cognition by telephone
DAN: dorsal attention network
DMN: default mode network
EVMI: episodic verbal memory (immediate recall)
EVMD: episodic verbal memory (delayed recall)
FC: functional connectivity/correlation
fMRI: functional magnetic resonance imaging
FPN: frontoparietal network
FS-FAST: FreeSurfer Functional Analysis Stream
GLM: general linear model
HC: healthy control
IPL: inferior parietal lobule
IR: inductive reasoning
LN: limbic network
LOC: lateral occipital cortex
MFG: middle frontal gyrus
mTBI: mild traumatic brain injury
MTG: middle temporal gyrus
OA: older adult
PCG: precentral gyrus
PCun: precuneus
PoCG: postcentral gyrus
PS: processing speed
RS: resting state
RSN: resting state network
SFG: superior frontal gyrus
SMN: somatomotor network
TBI: traumatic brain injury
VAN: ventral attention network
VF: verbal fluency
VN: visual network
WM: white matter
WMS: working memory span
YA: younger adult.
