# Supplementary material for: Functional Connectome Dynamics After Mild Traumatic Brain Injury According to Age and Sex
Source: Front Aging Neurosci. 2022 May 18;14:852990. doi: 10.3389/fnagi.2022.852990 (PMC9158471; doi:10.3389/fnagi.2022.852990)
Supplement: Supplementary file 1 [file Data_Sheet_1.docx]

Supplementary Material

# Supplementary Figures and Tables

## Supplementary Figures


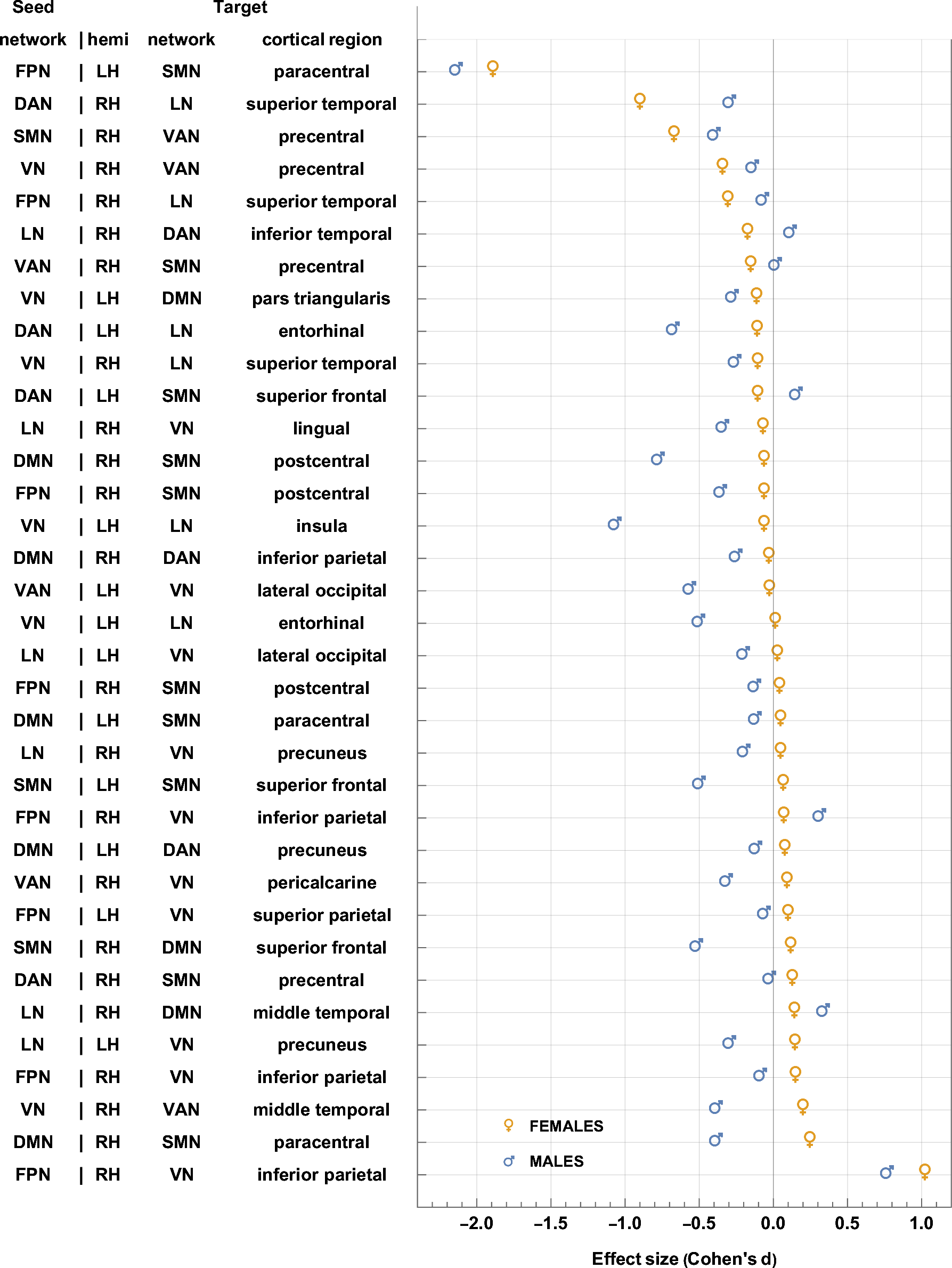


**Supplementary Figure 1.** Like **Figure 5**, with clusters sorted in ascending order by females’ FC changes. Abbreviation: FC = functional correlation.

## Supplementary Tables

----------------------------------------------------------------------

index d p seed target cortical clusters

----- ----------------- -------- ------- -----------------------------

area

---------------

local total

------- -------

M F ∆ network hemi network % cm^2^ cm^2^

----- ----------------- -------- ------- ---- -------- --- ----- -----

1 -2.14 -1.89 -0.25 0.0019 FPN LH SMN 99 11 12

2 -0.30 -0.90 0.60 0.0001 DAN RH LN 46 14 32

DMN 28 9

VAN 12 4

3 -0.40 -0.67 0.27 0.0012 SMN RH VAN 80 9 11

4 -0.14 -0.34 0.20 0.0110 VN RH VAN 60 5 8

SMN 33 3

5 -0.07 -0.30 0.23 0.0040 FPN RH LN 68 5 8

DMN 17 1

6 0.11 -0.17 0.28 0.0006 LN RH DAN 55 5 8

VN 45 4

7 0.01 -0.15 0.16 0.0285 VAN RH SMN 53 4 8

VAN 47 4

8 -0.68 -0.11 -0.57 0.0001 DAN LH LN 80 13 17

9 -0.28 -0.11 -0.17 0.0265 VN LH DMN 53 4 8

VAN 37 3

10 0.15 -0.10 0.25 0.0019 DAN LH SMN 90 11 12

VAN 10 1

11 -0.26 -0.10 -0.16 0.0001 VN RH LN 34 17 51

DMN 30 16

SMN 17 9

VAN 16 8

12 -0.34 -0.07 -0.28 0.0001 LN RH VN 97 45 47

13 -0.36 -0.06 -0.30 0.0001 FPN RH SMN 90 16 17

14 -0.78 -0.06 -0.72 0.0001 DMN RH SMN 51 16 31

VN 33 10

DAN 11 3

15 -1.07 -0.06 -1.01 0.0001 VN LH LN 34 20 60

DMN 28 17

SMN 18 11

VAN 13 8

16 -0.56 -0.03 -0.54 0.0001 VAN LH VN 97 32 33

17 -0.25 -0.03 -0.23 0.0046 DMN RH DAN 32 2 7

VN 31 2

DMN 26 2

FPN 10 1

18 -0.50 0.02 -0.52 0.0001 VN LH LN 59 17 29

DMN 38 11

19 -0.20 0.03 -0.23 0.0001 LN LH VN 74 72 97

DAN 10 10

20 -0.13 0.04 -0.17 0.0241 FPN RH SMN 100 8 8

21 -0.12 0.05 -0.17 0.0221 DMN LH SMN 100 8 8

22 -0.20 0.05 -0.25 0.0018 LN RH VN 83 8 9

DMN 14 1

23 0.31 0.07 0.24 0.0033 VAN RH VN 67 5 7

DMN 19 1

DAN 13 1

24 -0.50 0.07 -0.57 0.0001 SMN LH SMN 45 16 37

DMN 36 13

DAN 10 4

25 -0.12 0.08 -0.20 0.0096 DMN LH DAN 63 6 9

FPN 25 2

26 -0.32 0.10 -0.42 0.0001 VAN RH VN 100 25 25

27 -0.06 0.10 -0.16 0.0283 VAN LH VN 94 5 5

28 -0.52 0.12 -0.64 0.0001 SMN RH DMN 86 14 16

FPN 13 2

29 -0.03 0.13 -0.16 0.0355 DAN RH SMN 82 6 8

VAN 18 1

30 0.34 0.14 0.19 0.0135 LN RH DMN 62 4 7

FPN 37 2

31 -0.30 0.15 -0.45 0.0001 LN LH VN 73 16 22

DMN 25 6

32 -0.09 0.15 -0.24 0.0027 FPN RH VN 37 3 8

DAN 33 2

DMN 20 2

33 -0.39 0.20 -0.59 0.0001 VN RH VAN 26 39 148

DMN 25 37

SMN 19 28

LN 14 21

34 -0.39 0.25 -0.63 0.0001 DMN RH SMN 98 16 16

35 0.77 1.03 -0.26 0.0015 FPN RH VN 66 6 8

DAN 25 2

**Supplementary Table 1**. Like **Table 3**, with clusters in descending order of females’ effect sizes. Abbreviations: F = females, M = males.
